# Electronic structure and multiscale modelling of the hydroxyapatite–TiO_2_ bio-interface: from self-interaction error to predictive coating design

**DOI:** 10.1039/d6ra08442g

**Published:** 2026-10-09

**Authors:** Emaan Shahid, Harram Najum, Muhammad Tayyab Raza, Abdul Majid

**Affiliations:** a Department of Physics, Hafiz Hayat Campus, University of Gujrat Gujrat Pakistan abdulmajid40@yahoo.com; b Department of Physics, Gachon University 1342 Seongnam-daero, Sujeong-gu Seongnam-si Gyeonggi-do South Korea ali1214@gachon.ac.kr

## Abstract

Hydroxyapatite–titanium dioxide (HA–TiO_2_) heterojunction coatings are commonly applied to enhance the osseointegration of load-bearing orthopedic implants, but their clinical performance is limited due to coating delamination and fibrous tissue encapsulation at the implant–bone interface. Density functional theory (DFT) offers excellent pre-synthesis predictions on HA–TiO_2_-based designs, but self-interaction error (SIE) remains a major hurdle due to the well-known underestimation of the band gaps of TiO_2_ and oxygen vacancy formation kinetics. This leads to inaccurate predictions of localized oxidative stress, electron transport, and early-stage corrosion at the interface. A comprehensive review of the accuracy-cost trade-off of DFT+U, hybrid functionals (HSE06), and machine-learning interatomic potentials (MLIPs) for modelling the HA–TiO_2_ interface is given here. The electronic-structure corrections related to mesoscale phenomena accessible by molecular dynamics (MD) are also described. A systematic description of the stiff, ice-like hydration layer formed at the TiO_2_ surfaces is provided to shed light on the transport of Ca^2+^ and PO_4_^3−^ during biomineralization. Further, the dynamics of the hydroxyl dipoles, doping and nanostructuring that affect the nature and strength of the resulting interfacial adhesion energy are elaborated in detail. This multiscale approach that connects the accuracy of the electronic structure with the mesoscale hydration and ion-transport kinetics offers a physics-based, computationally informed path to the rational design of HA–TiO_2_ biomaterial coatings.

## Abstract 2

Understanding interfacial failure mechanisms in HA–TiO_2_ coatings to solve hydration and charge-transfer barriers: a review of multiscale modeling strategies bridging atomic physics with clinical osseointegration.

## Graphical abstract

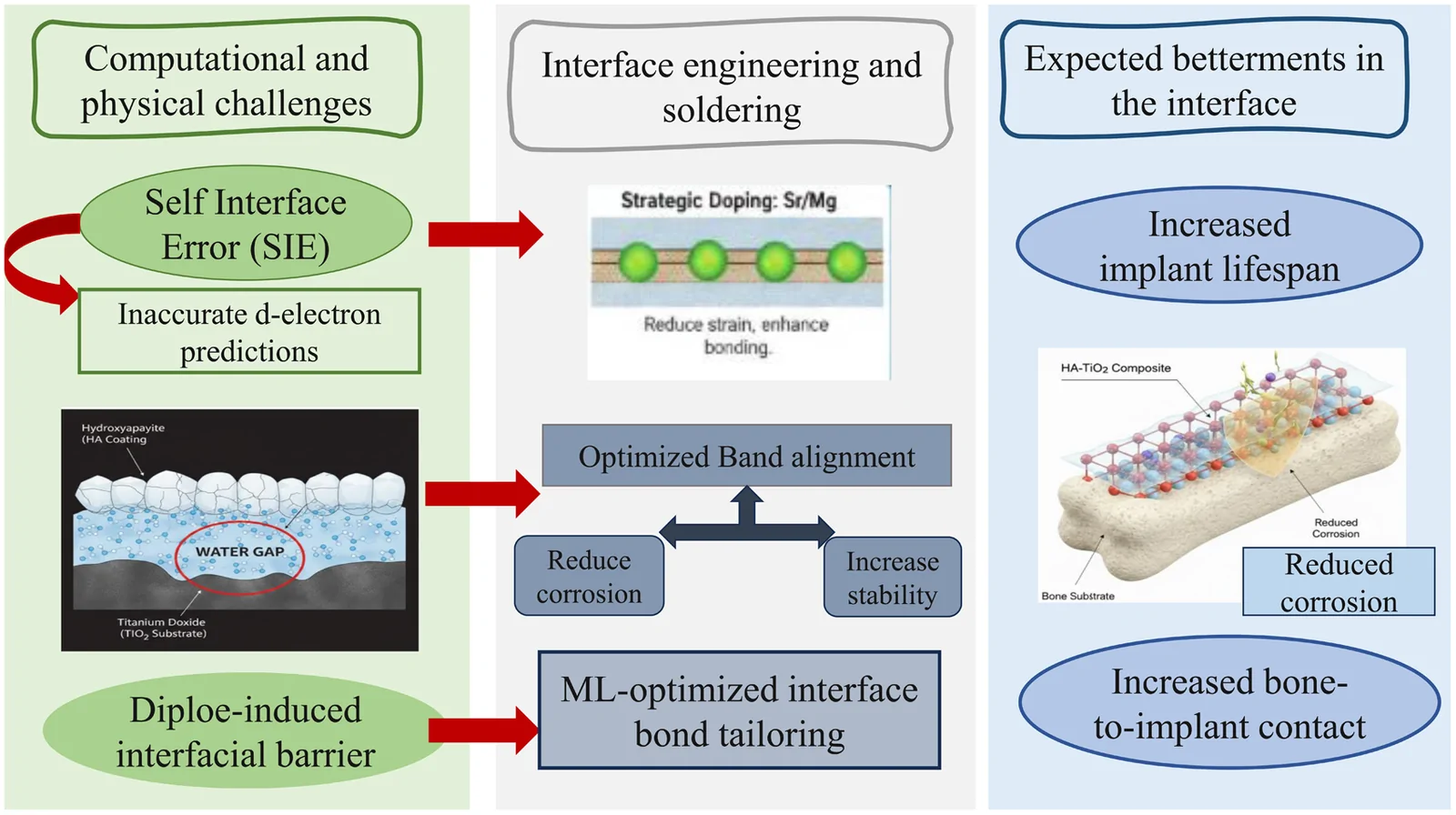

## 1. Introduction

Advances in orthopedic implants are being achieved by shifting the focus from the macroscopic mechanical stability to the electronic level.^1^ Typically, implant success has been judged by resistance to bulk mechanical stresses, which in too many cases may obscure the underlying complex, but potentially harmful, electrochemical activity taking place at the synthetic–organic interface.^1,2^ The long-standing problem of fibrous encapsulation, wherein the body recognizes the implant as foreign, stems from basic miscalculations in the electrostatics between the body's surface and the implant. Clinical attention tends to be given to the mechanical aspect of fixation, whereas the biological aspect of fixation is mediated by the quantum-level electronic aspect of the interface. Self-interaction error (SIE) is a major hurdle in predictive heterostructure optimization as typical DFTs tend to underestimate the band gaps of TiO_2_ and vacancy formation energies for oxygen, which can lead to inaccurate predictions of localized oxidative stress, electron transport, and early-stage corrosion at the interface. If electronic-structure methods do not adequately account for SIE, predicting surface reactivity is fundamentally problematic; therefore, coating designs may be flawed, for example by promoting electron trapping that could contribute to inflammatory responses. The focus shifts to subatomic events like hydroxyl dipole flipping, and the formation of a rigid hydration layer acts as a guard of biomineralization. Such a correlation of these nanoscale quantum events with continuum biomechanical models will offer a clear path towards overcoming current limitations in implantology. This shift depends on developing the HA–TiO_2_ heterojunction; in this case, structural fracture is not the primary concern, but the integrity of the interface between the synthetic material and the biological environment is key.^3^ It is a multiphase interface of inorganic transition metal oxides to organic bone tissue. In this architecture, the use of the mechanical scaffold of TiO_2_ coupled with the protection of the oxide layer (∼37 nm thick) renders the implant surface chemically quiescent in the aggressive environment with a high chloride content.^4,5^ In contrast, when other materials are applied, hydroxyapatite (HA) provides chemical similarity to bone mineral, reducing adverse biological responses and promoting osteoblast responses that facilitate implant integration. Rather, it is the atomistic physics at the point of contact, such as where localized electronic density is in contact with an ionic ceramic lattice, which influences the clinical outcomes.^6,7^

Previous reviews focused mainly on synthesizing and characterizing HA–TiO_2_ interfaces to evaluate their biological response. However, less focus has been observed towards computational modelling of the interfacial behavior, whose role is crucial in atomic-level understanding of the interfaces. Furthermore, the importance of SIE in DFT calculations, its effects on charge transfer processes, the hydration properties, and kinetic processes of corrosion reactions have not been systematically explored. Less focus has been given to the coupling between the multiscale phenomena of ion transport and the quantum-level electronic structure. The salient features of this review include a systematic assessment of the effects of SIE, the role of electronic structure methods (GGA/PBE, DFT+U, HSE06 and GW) in the calculation of formation energy of oxygen vacancies, band offsets, and reactivity at metal oxide interfaces. We identified important knowledge gaps in atomistic modelling, mesoscale modelling of hydration layers, modelling of selective ion transport rates, and piezoelectric coupling. Further, protocols on standardised physics-based benchmarks to bridge the quantum to clinical designs have been proposed. Hence, one novel contribution to complement the existing reviews would be to provide a computational guideline for rational design of HA–TiO_2_ biomaterial coatings by applying SIE correction, which upscales to the mesoscale hydration dynamics and downscales to translation to the clinic.

The electronic SIE, the structural water gap, and electrostatic barriers produced by a dipole are the three most difficult issues in high-performance implant development; that is why they are called the central challenges of biomaterials physics. This electronic blurring results in errors in the estimation of oxygen vacancy (V_o_) energies in electronic materials. Hence, careful computations are required to evaluate V_o_ energetics due to their role in initiating interfacial attachments and corrosion. If the charge density is not correctly mapped, the calculation of the adhesion energy *W*_ad_ may not be sufficiently accurate and may overestimate the strength of adhesion to the implant when changes in the physiological pH and ion loss occur.^8–12^ Physiological fluids, however, can form highly structured hydration shells on the surface of the TiO_2_ upon insertion, which are a critical physical barrier to prevent direct chemical bonding between the Ca^2+^, PO_4_^3−^ ions and the substrate, often resulting in coating delamination. In addition, the dipole alignment induced by the inversion of OH^−^ ions inside the HA lattice can act as an electronic shield to regulate the adsorption of proteins.^13–15^ These dynamics, if not addressed, may render the interface electrostatically inert, resulting in fiber encapsulation, as illustrated in Fig. 1. If these quantum-scale events can be simulated accurately, they could support predictive implant design in which atomic-scale features are tuned to favour integration.^16,17^

**Fig. 1:**
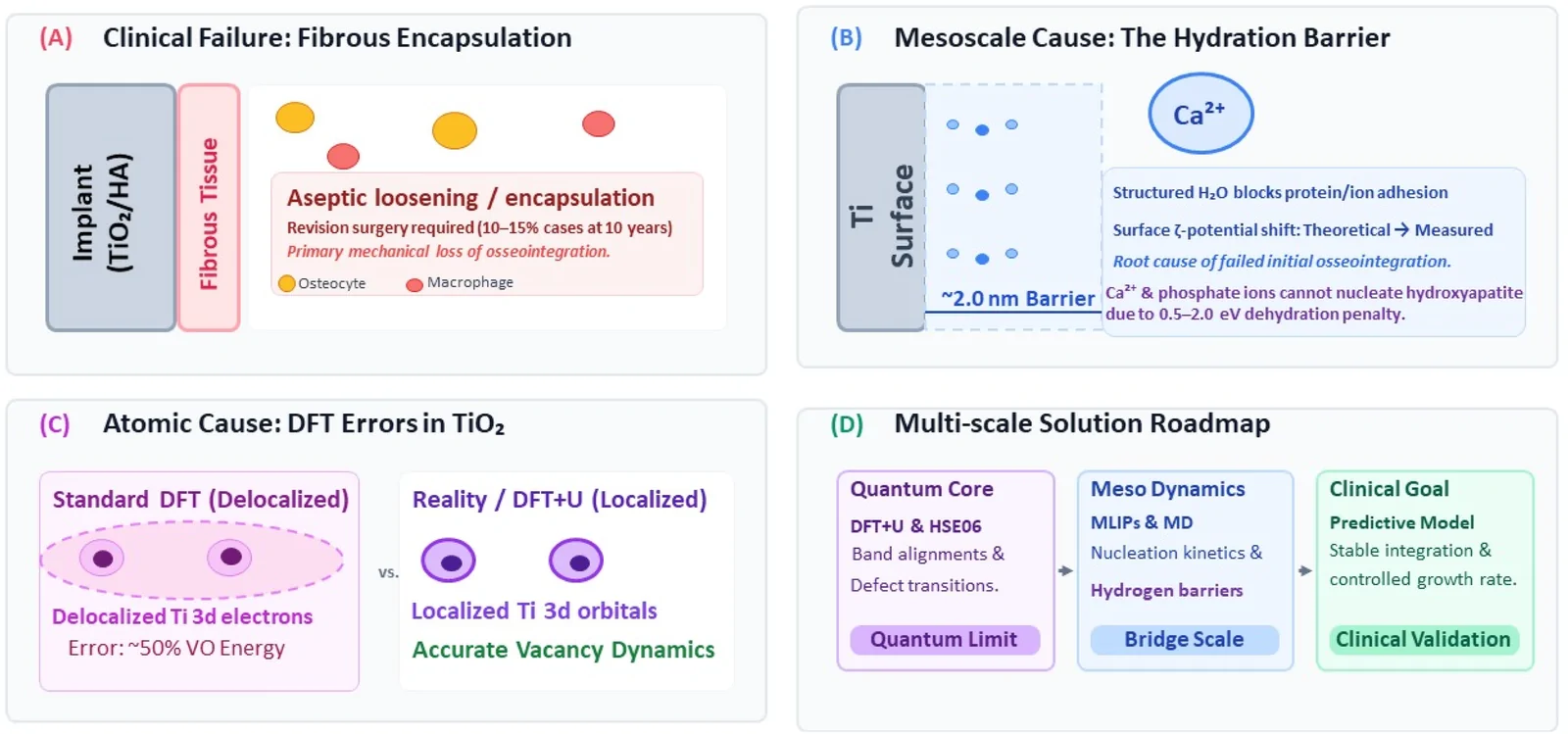
Schematic illustration of (A) HA–TiO_2_ implant failure *via* fibrous encapsulation, (B) mesoscale hydration barrier hindering bone nucleation, (C) atomic-level DFT delocalization errors, and (D) integrated multiscale modeling framework for predictive design.

## 2. Interface structure and biological barriers

### 2.1. The HA–TiO_2_ heterojunction

The hydroxyapatite and titanium dioxide (HA–TiO_2_) interface is a bio-ceramic heterointerface in which quantum size effects on electronics directly impact the biological response.^18^ Compared to the normal solid–solid interfaces investigated in materials physics, the HA–TiO_2_ interface operates between an inorganic semiconductor and a living tissue with active ionic ceramic under physiological conditions. At the interface level, the interface between the HA and TiO_2_ may be defined by the presence of dissimilar electronic structures.^19^ TiO_2_ is a transition metal-oxide semiconductor, and highly ionic crystalline hydroxyapatite is a wide-gap ionic insulator. Electronic redistribution occurs at the interface where the materials meet, forming local electrostatic fields and defect-mediated charge states. They are typically a concern in semiconductor physics, but are relevant to junctions and to surface reactivity, hydration dynamics, and affinity of biologically relevant species.^20,21^ As shown in Table 1, the HA–TiO_2_ interface is not necessarily a chemically or structurally abrupt interface. Instead, it grows into a strain-affected region having gradients of the compositions and concentrations of defects. This imperfect nature differs from the conventional assumption of computational interfaces, which are perfectly ordered, and reveals the relationship between structural disorder and electronic behavior.^22–24^

**Table 1:** Comparison of computational and experimental physical constants of TiO_2_ and HA

Material	Property	Experimental value	Standard DFT (GGA/PBE)	Advanced theory (HSE06/DFT+U)	Ref.
Rutile TiO_2_	Band gap	∼3.0 eV	1.88 eV	HSE06: 3.39 eV	25 and 26
Anatase TiO_2_	Band gap	∼3.2 eV	1.94 eV	HSE06: 3.60 eV	25 and 26
Brookite TiO_2_	Band gap	—	1.86 eV	HSE06: ∼3.3 eV	25
Hexagonal HA (*P*6_3_/*m*)	Lattice parameters	*a* = *b* = 9.430 Å, *c* = 6.874 Å	—	—	27
Rutile TiO_2_ (110)	Surface energy	Not reliably measured	∼0.48 J m^−2^	∼0.98 J m^−2^ (DFT+U)	28
Hexagonal HA (*P*6_3_/*m*)	Band gap	∼7.4–7.7 eV	5.23 eV	—	29
Rutile TiO_2_	V_o_ formation energy	—	Condition-dependent: VO^2+^ is most stable under n-type, O-poor conditions	Condition-dependent	30 and 31
Anatase TiO_2_	V_o_ formation energy	—	Condition-dependent	Condition-dependent	30 and 32
Rutile TiO_2_	Bulk lattice constants	*a* = *b* = 4.5937 Å, *c* = 2.9581 Å	—	—	33
Anatase TiO_2_	Bulk lattice constants	*a* = *b* = 3.7842 Å, *c* = 9.5146 Å	—	—	34 and 35
Rutile TiO_2_	Hubbard U (Ti 3d)	—	*U* ≈ 3–5.9 eV (varies)	*U* = 5 eV gives *E*_g_ ∼3.12 eV	35 and 36

### 2.2. Hydroxyl dipole dynamics in HA

Hydroxyl groups on the HA surface play a critical role in triggering biomineralization *via* hydroxyl flipping. The energy landscape of these flips has been systematically investigated using first-principles calculations. Kasamatsu and Sugino identified several ion migration mechanisms in HA, including OH^−^ flipping with activation barriers of 0.2–0.8 eV and interstitial proton hopping with significantly lower barriers of 0.025–0.25 eV.^37^ The latter mechanism, proton transfer between neighbouring OH^−^ groups *via* the Grotthuss mechanism, is considerably faster than the full OH^−^ reorientation. *Ab initio* molecular dynamics (AIMD) simulations show that flipping events of OH^−^ can be observed only at high temperatures. MD simulations at 1000–1500 K have been demonstrated to witness flipping events in realistic simulation timescales because the 0.2–0.8 eV barriers are too high to allow flips at room temperature.^37^ This is consistent with machine-learning potential simulations that show that flip-flop reorientation of HA starts at a temperature around 473 K.^38^ The non-random behavior of hydroxyl dipoles is important for understanding the bioelectrical performance of the HA surface, as shown in Fig. 2.

**Fig. 2:**
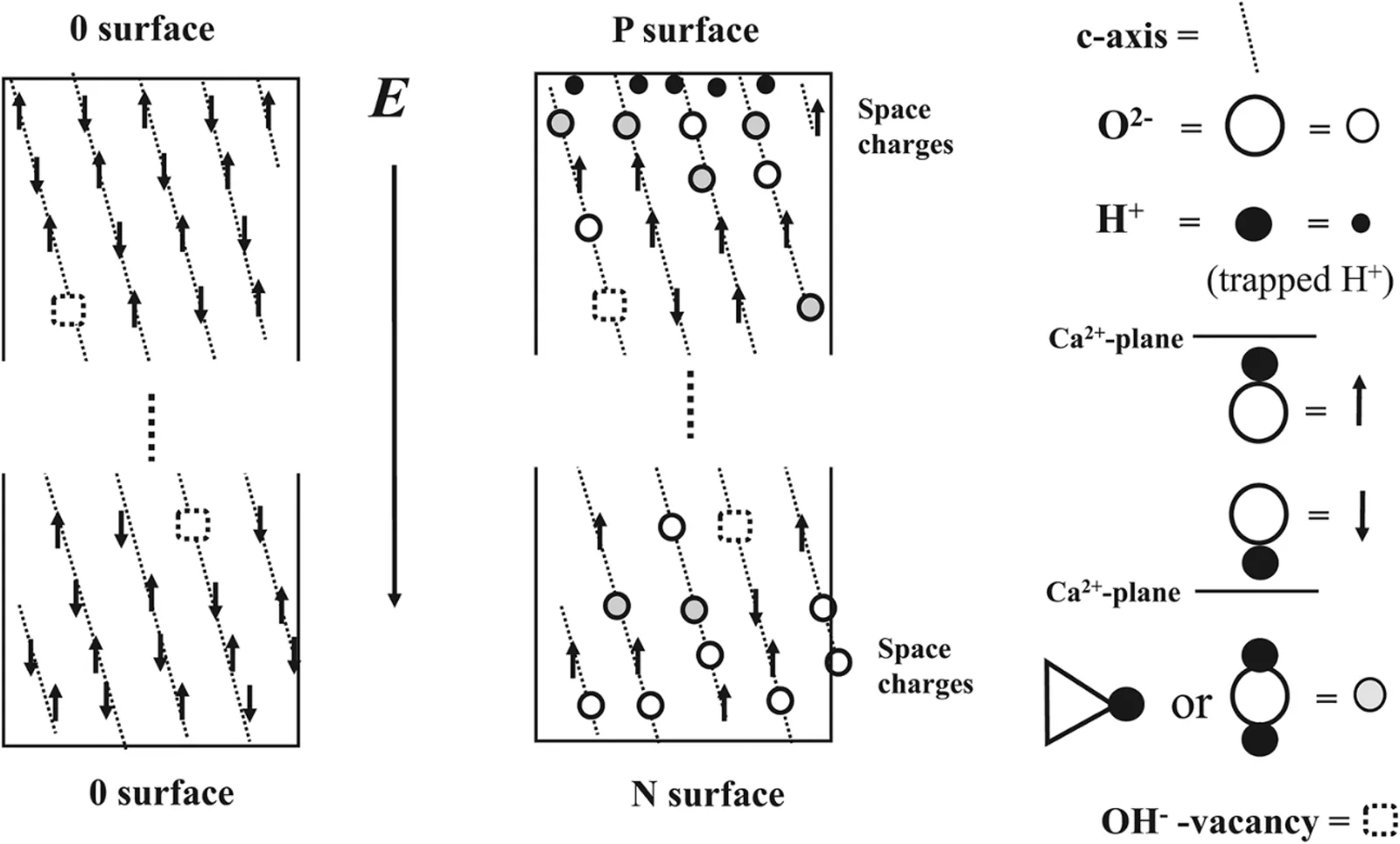
Hydroxyl dipole orientation in HA: randomly at rest and aligned under an electric field, driving ion migration and space-charge formation. Reproduced with open access permission from ref. 41. Published under a License CC BY-NC-ND 4.0.

Mechanical stress can generate a potential local electrical field at the HA–TiO_2_ interface when the physiological load is applied. This field aligns the non-collinear hydroxyl dipoles to lie along an axis parallel to the *c*-axis of the HA crystal, which can produce a net polarization. This ordering may influence the electrostatic properties of the interface. The resulting dipole alignment is important because the migration of protons and other charge carriers toward the grain boundaries, which can contribute to space-charge polarization, a possible mechanism of the piezoelectric coupling leading to osteoblast responses. In the living organism, dipole reorientation may influence local electrostatic signals of the implant, and the rapid dipole reorientation modulates the local zeta potential, which may be sensed by osteogenic cells.^39,40^ These dynamic dipole structures, as far as the bio-interface is concerned, generate spatially heterogeneous surface-charge distributions, which have been attributed to accelerated calcium absorption. Laboratory electrostatic maps indicate that the maximum swings of the local surface potential can be elevated by flipping to 50 mV, and that sites favourable for calcium-phosphate nucleation are where this difference attains significant values. The fluctuations in these charges serve as electrophoretic anchors to Ca^2+^, potentially increasing mineral nucleation rates.^38,40^

Mesoscale kinetic Monte Carlo simulations, constructed using the atomistic flip rates, prove the existence of such dynamic behavior that is indeed accelerating nucleation. The existence of rapid reorientations of hydroxyl on the surface is supported by surface-sensitive approaches like sum-frequency generation (SFG) vibrational spectroscopy and has timescales on the same order as those in simulations. The study in which HA surfaces are designed to ensure that their hydroxyl groups are in dynamic motion portrays a 15–25% hump in early mineral deposition, demonstrating the functional significance of the flip in the dipole.^42,43^ It is to this day difficult to get the entire picture of how dipole flips, hydration, and ion transport will all interact, particularly in the complexity of the physiological environment. However, the present hybrid DFT, AIMD, and mesoscale modelling act as a good base for interpreting the dipole flip mechanism to fabricate improved implant surfaces that can be better integrated into the bone.

## 3. Charge transfer and errors

### 3.1. The self-interaction error in TiO_2_

Conventional DFT approximations face challenges in correctly handling the SIE due to inadequate d-electron localization; thus, it is inherently difficult to describe the TiO_2_ surface, and specifically the HA–TiO_2_ interface.^44^ Similar DFT-based studies of dental implant materials have also highlighted these limitations for evaluating mechanical properties and structural stability.^45^ SIE produces delocalization of d-electrons in titanium atoms, which causes inaccurate results of the electronic structure, defect energy, and surface chemistry that are crucial in the implant behavior. This delocalization error affects both the V_o_ formation energies and the computed bandgap.^46,47^ This error propagates into the formation energies and localization of charge of oxygen vacancies (V_o_) as active sites of corrosion and surface reactivity in physiological systems. Physiologically, the vacancies of oxygen act as catalytic centers producing reactive oxygen species (ROS). The inability to correct SIE may overestimate implant-induced oxidative stress, which may influence inflammatory responses and fibrous encapsulation.^17,47^ The improper localization of the d-electrons also hinders the proper description of polaron formation and charge trapping on the TiO_2_ surface, which determines the electron transfer process necessary to protect surfaces against corrosion and facilitate biocompatibility.^48^ In a biomedical perspective, the importance of the accurate modelling of oxygen vacancies lies in the fact that such defects regulate the charge of a surface and the adsorption of proteins, and define the electrochemical stability of implants throughout the healing tissues.^17,49,50^

Underestimating V_o_ formation energies can lead to false predictions of high vacancy concentrations, potentially suggesting increased corrosion propensity. On the other hand, the correct localization of state defects is consistent with passivation as observed experimentally. Oxygen vacancies on TiO_2_ surfaces play a dual role in reactive oxygen species (ROS) chemistry: under photocatalytic or electrocatalytic conditions, they act as active sites that promote O_2_ activation and ROS generation;^51,52^ however, once passivated or stabilized by surface reconstruction, vacancy-based electron trapping can suppress excessive ROS formation. Recent multiscale models have coupled kinetic Monte Carlo and continuous electrochemical models with corrected electronic structure to predict multiple degradation surfaces of implants under physiological conditions, and these models have demonstrated that enhanced description of electrons and vacancy energetics would improve predictions of corrosion onset to within 30%.^53^ The estimation of lifespan is affected by the error.^49,54,55^ The predictive reliability of the V_o_ behavior in TiO_2_ modelling and, accordingly, the rise of the corrosion resistance and biocompatibility of the implant depend on the proper treatment of SIE. The future of predictive models lies in the combination of state-of-the-art electronic structure techniques with experimental validation, which would serve as the foundation for future technology for the design of the next generation of biomedical interfaces. Table 1 compares the values of physical constants for HA and different forms of TiO_2_, as well as experimental, DFT, and advanced DFT-based studies, to show the differences that occur in the HA–TiO_2_ interface with the changes.^56,57^ Therefore, the SIE is the main computational challenge which must be addressed before analytical modelling of HA–TiO_2_ interfaces because it influences the calculations of charge transfer, band alignment, and hydration dynamics.

### 3.2. Interfacial issues during charge transfer

The findings from intensive computational studies on the HA–TiO_2_ interface have shown that interfacial charge transfer and band alignment have complexities in accurate modelling of the interfaces that determine the bioactivity of implants.^49^ The accuracy of these predictions is strongly dependent on the correct handling of the SIE in the DFT calculations, as discussed in Section 3.1. The band offset at the interface has been investigated using an integrated approach combining DFT and GW perturbation theory.^58,59^ The calculated band offsets revealed type II staggered band alignment with valence and conduction band levels in respective ranges of 0.65–0.85 eV and 0.45–0.70 eV to allow spatial separation of charge carriers.^58,59^ The study on interfacial charge transfer points to charge redistribution in the range from −0.016 to −2.429 e^−^, depending on the termination and stacking configuration, with variations in local electrostatic potential between 0.1–0.3 V across the interface.^60^ The localized defect states at the interface, with oxygen vacancies in TiO_2_ and hydroxyl termination of HA, influence the chemical processes related to electron transfer. The oxygen vacancies create mid-gap states, acting as deep levels or electron traps, at the energy position of 2.0–3.5 eV below the conduction band edge.^30,32^ The proton-coupled electron transfer (PCET) pathways have been reported in which the intrinsic water molecule at the interface between the solid and solution phases of the catalyst plays a role in controlling the redox reaction.^61,62^ The experimental electrochemical impedance spectroscopy (EIS) validates the computational findings, where surface hydroxylation of HA-coated TiO_2_ implants leads to low charge transfer resistance with current densities of 0.1–10 µA cm^−2^.^63^ Similar results have been obtained from photoelectrochemical experiments, according to which the band offsets can be tuned *via* surface functionalization to modulate ROS chemistry—enhancing ROS-mediated antimicrobial activity under photocatalytic conditions,^64^ while suppressing excessive ROS generation after surface passivation. The activation energy barriers for interfacial charge transfer have been determined in the range of 0.2–0.5 eV, and the adhesion energy of 1.43–1.66 J m^−2^ for the bare interface directly affects the electrochemical stability of the implant surface.^60^ The large-scale models, which also involve continuum electrochemical models, are related to the simulation of prototypes on long-term diffusion-dominated corrosion behavior in physiological environments. For instance, kinetic Monte Carlo with charge transfer rates determined by DFT, giving the proper band alignment and localization of defects, can predict larger ranges of corrosion and durability measurements.^65^ However, there are a few obstacles in capturing the dynamic restructuring of the interfaces in combination with the mechanical stress and biochemical environment on the electronic properties.^49^ These point to the value of advanced approaches including time-dependent DFT and machine-learning potentials, which can enable longer-timescale simulations with electronic accuracy.

The mechanistic knowledge of the charge transfer event involved in the operation of implants includes parameters such as band offsets, electron transfer rates, and energetics of interfacial defects determined using computational studies.^63^ However, it is useful to link these findings with experimental validation and multiscale modelling that sheds light on the value of predictive design of biomaterial interfaces with high corrosion resistance and bioactivity. It offers useful strategies to study the implant surface, which is electrochemically inert but biologically active to allow bone repair.^66^

### 3.3. Fibrous encapsulation

Fibrous encapsulation remains a common failure mechanism in orthopedic and dental implantology despite improved understanding of biomimetic surface engineering. Experimentally, fibrous tissue development can be explained in terms of biological processes governed by fundamental principles in such a way that its driving forces lie in interfacial quantum phenomena.^67^ The computational predictions offering insights into electronic band alignment, charge transfer, and defect states at the HA–TiO_2_ interface shed light on electrochemical instability and ion flux dissociation, to improve understanding of the integration of fibrous over osseous tissue.^68^ The utilization of integrated experimental and computational approaches indicates that the neglect of the interfacial effects produces false observations of fibrous encapsulation dynamics. The direct interfacial charge redistribution influences protein adsorption, osteoblast adhesion, and mineralization. The experimental evidence based on probe force microscopy is capable of identifying the local potential changes of 100–150 mV related to HA–TiO_2_ surfaces, which involve changes in osteogenic protein conformations and cell adhesion.^69^ The DFT calculations make energetic predictions on V_o_ in TiO_2_ to shed light on the mid-gap electronic states, which are capable of trapping electrons and altering the local surface reactivity.^70^

The computational predictions supported by electrochemical impedance spectroscopy (EIS) and potentiodynamic polarization indicated that current densities in the range of 0.1 and 10 µA cm^−2^ have a direct relationship with the intensification of fibrous encapsulation. At the same time, dissolution rates of Ca^2+^ and PO_4_^3−^, measured using inductively coupled plasma mass spectrometry (ICP-MS) and determined at rates of 10^−1^ to 10^−9^ mol m^−2^ s^−1^, cause local pH changes, further worsening inflammatory reactions.^71^ It is proposed that V_o_ at the TiO_2_ surface traps electrons to change the local surface charge, resulting in a corrosive microenvironment, as shown in Fig. 3. The defect passivation strategies such as doping or nanostructuring are therefore essential to suppress this phenomenon. This leads to pH fluctuations and increased current densities (0.1–10 µA cm^−2^), which together alter the biological response toward fibrous encapsulation rather than bone formation.^72^

**Fig. 3:**
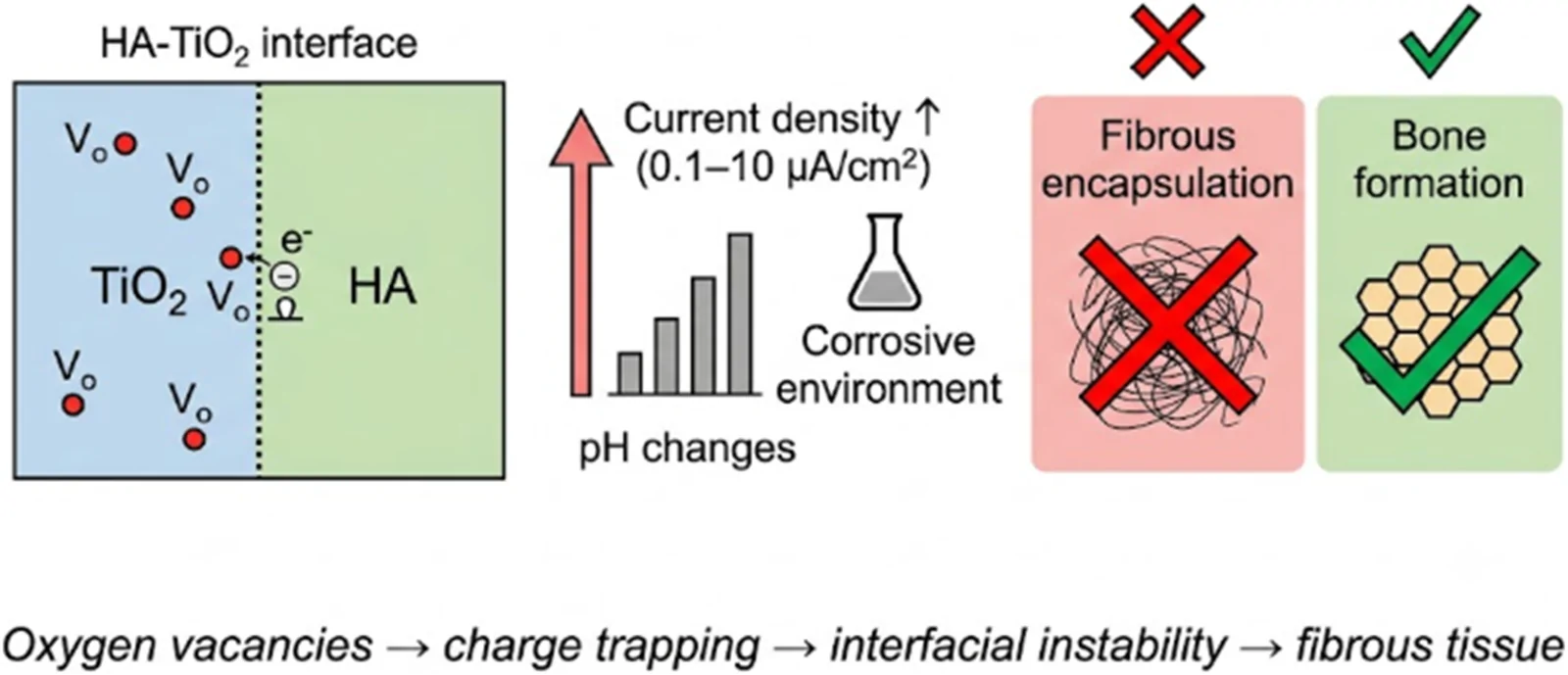
Visual representation of oxygen vacancies at the HA–TiO_2_ surface leading to charge trapping and fibrous encapsulation.

Another determinant of implant integration at a quantum level is called interfacial hydration. XRR and MD simulations have shown that the two uppermost layers of the hydration layers on TiO_2_ surfaces are organized into ice-like structures that compete for adsorption sites. Traditional DFT tends to underestimate van der Waals forces within such hydration layers, and it thus does not give correct predictions of HA nucleation kinetics.^65,71^ The proposed mechanism responsible for fibrous entrapment, such as charge trapping by defects and interfacial electronic mismatches and hydration-layer competition, is thought to necessarily be a quantum-scale effect, which has been discussed in previous sections. The experimental evidence analysis and multiscale simulations offer practical information on the field of defect passivation, surface functionalization, and nanoscale engineering to position the HA–TiO_2_ interface in a paradigm where a single carrier of macroscopic clinical outcomes is the processes at angstrom and femtosecond scales.^73^

## 4. Mechanical and chemical engineering of the interface

### 4.1. Lattice mismatch and strain problems

The degree of lattice matching of HA (001) and TiO_2_ (110) surfaces, which is made possible by the effective epitaxial mismatch, results in a quantitative lattice matching with a lattice mismatch of approximately 3.2–4.8%.^74^ The slabs of amorphous HA and TiO_2_ are shown before and after relaxation in Fig. 4, where the interface is found to be stable, and there is no lattice mismatch. Calculations with the GGA+U method have been performed to obtain a small fraction (0.15–0.28 eV per 1% strain) of the increase in the conduction band minimum of the bandgap when tensile strain is applied to TiO_2_(110). Further, a decrease in the valence band maximum (0.12–0.22 eV per 1% strain) has been observed upon application of the compressive strain. Calculations indicate that strain-induced Fermi level shifts resulting from the Ti 3d states are enhanced by strain and favor of the strain-induced degeneration of the Ti 3d orbitals to effectively 2D states. This degeneration leads to anisotropic electronic dispersion and a decrease in the in-plane electron effective mass up to 15%, corresponding to an increase in lateral carrier conductivity at the interface. The indication that strain relaxation by formation of misfit dislocations at approximately a critical thickness (higher than natural object thicknesses) at approximately a critical thickness of approximately 2 nm can alleviate local elastic strains by a factor of 30% implies that the formation of misfit dislocations contributes to the introduction of electronic states halfway between bands (associated with Ti interstitials and oxygen vacancies) at trap depths of about 0.3 eV.^70,75^ Local lattice distortions of up to 5% that are observable with GPA analysis of HRTEM images are shown to vary linearly with an increase in interfacial energy density between values of the order of 0.5 and 1.2 J m^−2^.^76^ The calculation of adhesion energy with the use of DFT shows that a moderate tensile strain change improves the value of energy up to 1.6 J m^−2^ at the strain adjacent to the basis point. However, beyond strains near 4.5%, about a 20% decrease in *E*_adh_ is observed as a result of defects and electronic trapping, which promotes interfacial instability owing to cyclic shear stress of density near 1.5–2 MPa, equivalent to physiological loading.^12^ The general results of both studies indicate that epitaxial strain is a significant control strategy in the management of DOS modulation, defect formation and mechanical stability at the interface of HA–TiO_2_.^9,74^

**Fig. 4:**
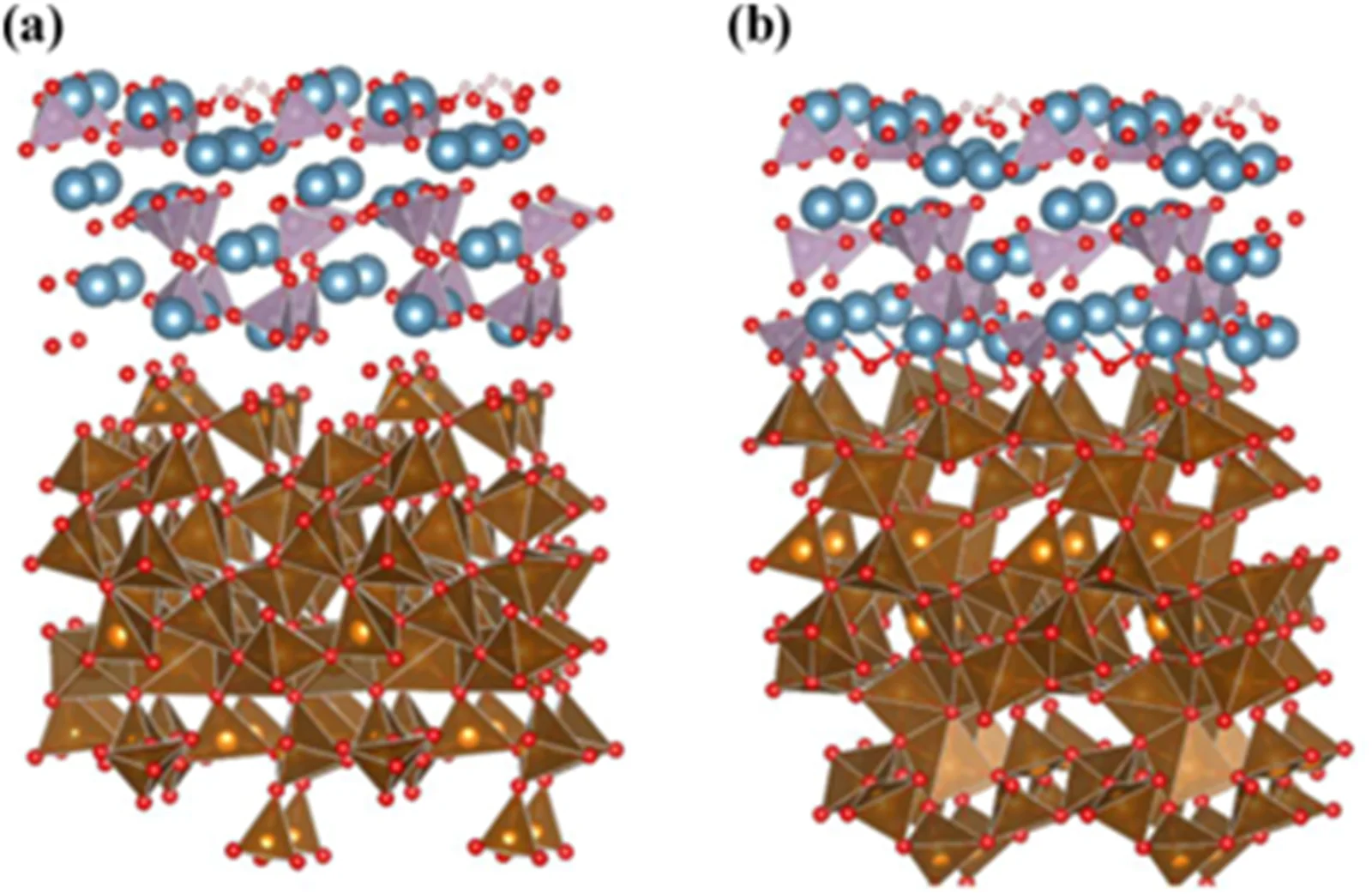
Slabs of the stacking model for a HA and TiO_2_ interface (a) before and (b) after relaxation, where the strain is accommodated.^60^ Reproduced with permission from the American Chemical Society.

Thermodynamic analysis (*ab initio*) shows that the lowest point in the free energy of the strained interface shifts to moderately tensile structures at physiological temperatures, which suggests that moderate tensile strain may be energetically favourable in the growth of the coating as well as annealing after deposition. The strain gradients at the interface generate intrinsic piezoelectric-like polarization fields within warped TiO_2_ octahedra and generate localized electric fields of the order of 10^7^ V m^−1^, which cause further perturbation of the local DOS at the Fermi level.^3,77^ A charge density difference tensile strain map indicates that tensile strain enhances interfacial electron accumulation, particularly at the bonding junctions, and strengthens mixed ion–covalent interactions. The strain-induced softening of low-frequency interfacial modes can be performed by phonon density of states and generates more vibrational entropy, resulting in the thermodynamic stabilization of strained interfaces.^11,78^ These electronically and vibrationally mediated strain effects slow down the nucleation of cracks during cyclical mechanical loading by increasing energy barriers to interfacial movement and causing significant energy barriers of approximately 0.203 eV. The electromechanical response obtained through regulated epitaxial strain, in turn, determines short-term electronic variability and the long-term stability of the HA–TiO_2_ implant interfaces.^43,79^

### 4.2. The space-charge layers for the charge transfer

The DFT hybrid functional (HSE06) calculations on valence-band offsets (∼0.65–0.85 eV) and conduction-band offsets (averaging 0.45–0.70 eV) in the HA(001)/TiO_2_(110) interface indicate that, on average, a type-II staggered interface is expected between the two interfaces with valence- and conduction-band offsets. This band alignment can promote directional charge transfer across the interface, and the net electron transfer densities of order 1.5–2.3 × 10^−2^ cm^−2^ are obtained using Bader charge analysis.^80–82^ The widths of space-charge layers can be self-consistently simulated by Poisson–Schrödinger simulations at approximations of 2–5 nm in TiO_2_, and space-charge layers can build up potentials of up to 0.3 V with physiological-like dielectric conditions.^83,84^ The conduction band offset is made 0.1 eV larger by tensile strain, making the electron confinement within TiO_2_ and thereby reducing interfacial recombination, and is decreased by compressive strain, inducing back-transfer of carriers. The result of PDOS indicates that Ti 3d orbital states are the dominant electron-accepting states; HA has Ca 3d and O 2p orbital states, which are the dominant electron-donating states within the cell, which agrees with the results of UPS and XPS, which indicate high vacuum level shifts (of Ti 3d and O 2p) of 0.2–0.4 eV.^85^

The electronic structure alone is not sufficient to explain that local electric fields can be increased by approximately 40% using the difference in dielectric (*ε*) of TiO_2_ (with *ε* ≈ 80) and HA. Such interfacial defects contain oxygen vacancies and phosphate vacancies and serve to introduce mid-gap states around the conduction band edge with an energy of approximately 0.25–0.35 eV that effectively screens the space-charge field in a local area and also reduces band bending.^8^ According to SKPM measurements, these effects are supported by the fact that a variation in a range of approximately 80 mV may exist over nanometer-scale regions. This regulated electrostatic landscape controls protein adsorption energetics and cell surface interactions between electronic band offsets, directly proportional to cell and implant adhesion in cyclical mechanical deformations, and corrosion resistance of osteoblasts.^81^

### 4.3. Doping for reducing the interfacial energy

Divalent cation interfacial doping of HA with Sr^2+^, Mg^2+^, and Zn^2+^ is an efficient mechanism for lowering interfacial energy and increasing chemical bonding at the HA–TiO_2_ interface. Fig. 5 represents the before- and after-relaxation structures of the HA–TiO_2_ interface, where HA is doped with Si to enhance the adhesion of the interface.^86,87^ Calculation of Hubbard *U* corrections shows that the interfacial formation-energy barrier decreases by approximately 12–25% relative to the undoped system, as Sr-doping produces the most significant stabilization, reducing the interfacial formation-energy barrier down to −0.35 eV per atom of dopant.^87,88^ Local lattice deformations caused by ionic-radius mismatch can be measured by changes in bond lengths of approximately 0.05–0.12 Å, which increases the orbital overlap across the interface. PDOS study demonstrates that there is intensive hybridization between Sr 4d and Ti 3d states, resulting in the enhancement of localized interfacial charge density by approximately 15–20% in the area of the dopant of about 3 Å.^16,89^

**Fig. 5:**
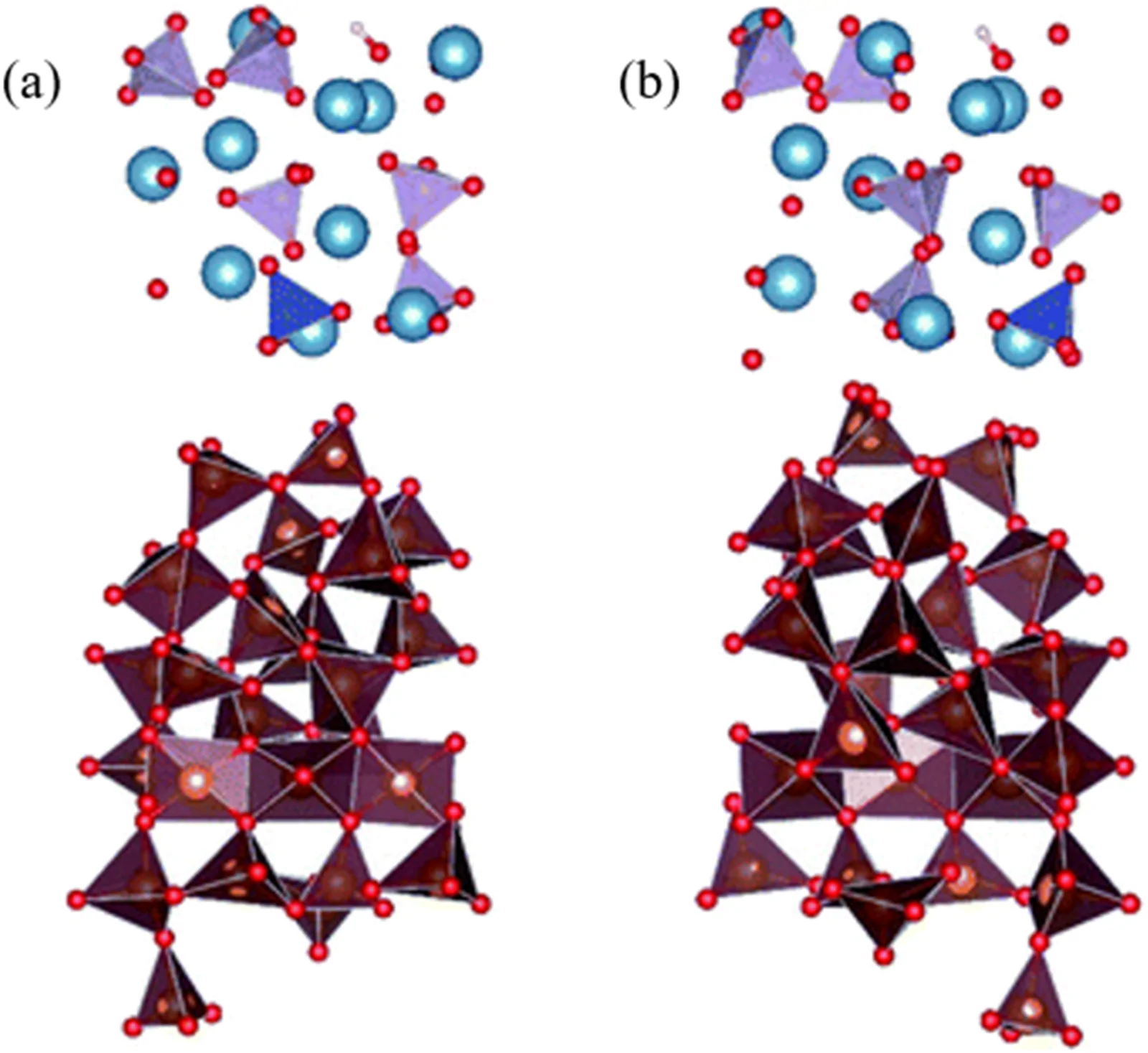
Structural models of Si-doped HA and TiO_2_ interface (a) before and (b) after relaxation. Reprinted from ref. 12 with open access permission from the Royal Society of Chemistry.

The Mulliken population analysis shows an increase in charge transfer, which decreases the interfacial dipole moment and decreases the electrostatic potential barrier. Thermodynamic integration calculations show that the interfacial energy density, which is about 1.1 J m^−2^ in the unperturbed system, decreases to about 0.75 J m^−2^ at a concentration of about 5% Sr and a 20% decrease in the energies of the defects formed.^90,91^ At 300 K, MD simulations indicate that doped interfaces can withstand shear forces up to about 2 MPa, whereas undoped interfaces can withstand pressures of about 1.3 MPa, which is deduced to be associated with mechanical integrity. Electrostatic modelling is expected to predict that Sr-doping raises the surface potential by approximately 0.15 V, increases the electrostatic attraction with osteoblast membranes with a negative potential, and raises the force of adhesion by about 18–25%.^92^ The dopants Mg and Zn have smaller yet significant charge-to-density and surface potential, and Zn has also created shallow states of the valence band, which can support antimicrobial activity. All these dopant-introduced lattice distortions, charge redistribution, and electrostatic modulation, in general, are chemical interactions which reinforce the interface and enhance long-term biomechanical function.^13^Table 2 provides various characteristics of the HA–TiO_2_ interface that change when various alterations are undertaken on it in an attempt to improve its functions in the medical field.

**Table 2:** Comparison of structural properties of pure and modified HA–TiO_2_ interfaces.^12^

System	Functional	Stacking	*W*_ad_ (J m^−2^)	Δ|*W*_ad_|^a^ (%)	Physical insight
a-HA/a-TiO_2_	PBE	I	−0.690	—	Stable interface with covalent bonds
a-HA/a-TiO_2_	PBE	II	−2.030	—	Stronger interaction than stacking I
a-HA/a-TiO_2_	DFT-D3	I	−1.740	—	vdW interactions contribute significantly
a-HA/a-TiO_2_	DFT-D3	II	−0.930	—	Weaker interaction with vdW correction
a-Si-HA/a-TiO_2_	PBE	I	−1.370	+99	Si doping improves adhesion
a-Si-HA/a-TiO_2_	PBE	II	−2.855	+41	Highest adhesion strength among models
a-Si-HA/a-TiO_2_	DFT-D3	I	−0.870	−50	vdW effects modify Si-doped interface
a-Si-HA/a-TiO_2_	DFT-D3	II	−2.315	+149	Strong adhesion with vdW correction

a Relative change in the magnitude of the work of adhesion caused by Si doping, Δ|*W*_ad_|, evaluated for the same functional and stacking. A more negative *W*_ad_ indicates stronger adhesion. Ref. 12 quotes the PBE increases as about 50% (stacking I) and 30% (stacking II) because it normalises by the doped value.

## 5. The wet environment: dynamics of the interface hydration layer

A major challenge in predicting implant behavior is transferring a prosthetic material from ultra-high vacuum modelling conditions to the complex physiological environment. The wet physics of the HA–TiO_2_ interface is analyzed in this section, which involves the formation of the hydration shield, followed by the thermodynamic barriers to protein adsorption.^93^ By coupling the electronic understanding of the interface with the fluid mechanics of the biological environment, a predictive framework for the early steps of osseointegration is being developed. This interfacial study is essential in the context of the modulation of the competition between interfacial water molecules and osteogenic ions by nanoscale topographies and surface charge densities.^94^

### 5.1. Water adsorption and the hydration layer on the TiO_2_ surface

The initial events during implantation involve the interaction of water with the TiO_2_ surface. Table 3 summarizes the water adsorption energetics, dynamics of adsorption, and mechanistic pathways of adsorption on TiO_2_ surfaces. Experimental and computational studies have established that the anatase TiO_2_ (101) supports both molecular adsorption and dissociation, with binding energies of approximately −0.73 eV.^10,95,96^ On the other hand, some experimental studies show that molecular adsorption on rutile TiO_2_ (110) is largely molecular, with partial dissociation at defect and step sites.^15^ These locations are essential since they determine the manner in which the hydration layer reorganizes to enable bone minerals to bind. In comparison, computational models predict that TiO_2_ nanotubes have a more diverse distribution of adsorption energies that are curvature- and site-dependent, generating geometry-modulated molecular and dissociative adsorption.^97,98^ The region of the implant surface in contact with the physiological environment is controlled by a structured hydration layer, which plays a key physical gatekeeper role in bone repair.^96^ It is a multilayered and multidimensional zone of transition, rather than a simple border interface. DFT calculations suggest that, based on theoretical considerations related to the deprotonation of the surface –OH and –H_2_O groups, the oxide surface electronic structure-*i.e.* the localization of d-electrons and the presence of oxygen vacancies-determines the dominant form of adsorption. Adsorption occurs in the hydrogen-bonding mode on a stoichiometric surface, but in the presence of vacancies, dissociative adsorption takes place, causing the water molecules to dissociate into surface hydroxyls.^15,99^ Computational models predict that this process forms a high-density, 2 nm thick layer of hydration whereby the water molecules have a quasi-solid, ice-like structure rather than bulk liquid behavior. To put these values into perspective, the mean thermal energy at physiological temperature is found to be approximately 0.026 eV.

**Table 3:** Water adsorption energies on surfaces of TiO_2_

Surface state	Reported adsorption energies	Dynamics/simulation	Ref.
Flat TiO_2_ (rutile 110)	Water adsorption binding energy −0.41 eV (−40 kJ mol^−1^)	Molecular adsorption, dissociation fraction of 22%	95
		The lifetime of the hydroxyl of the organometallic complex is about 7.6 ns	
Flat TiO_2_ (anatase 101)	Water adsorption binding energy −0.73 eV	A combination of molecular and dissociative processes	128
		H_2_O and OH species exist in a state of dynamic equilibrium	
TiO_2_ nanotubes	∼0.3–0.8 eV surface curvature depends on specific sites	Geometrical locations and interactions are favoured	97
		Site-specific dynamics	

The calculated binding energy of these molecules is about 29–42 times higher than the thermal energy according to DFT findings. AIMD simulations and phase-sensitive SFG spectroscopy were used to investigate the orientation and hydrogen-bonding structure of interfacial water on the TiO_2_ surface, as shown in Fig. 6. The spectral localization at different depths indicates that the topmost surface layer of water is highly oriented toward the TiO_2_ surface, followed by the next layer oriented toward bulk water. The contribution of chemisorbed hydroxyl groups and H-bonded OH groups to the SFG response is different, which helps to understand how surface hydroxylation and water orientation create the superhydrophilic behavior of TiO_2_. These simulations provide novel insights into the molecular basis of the physics of SFG intensity spectra, verified by the reconstructed SFG intensity spectra.

**Fig. 6:**
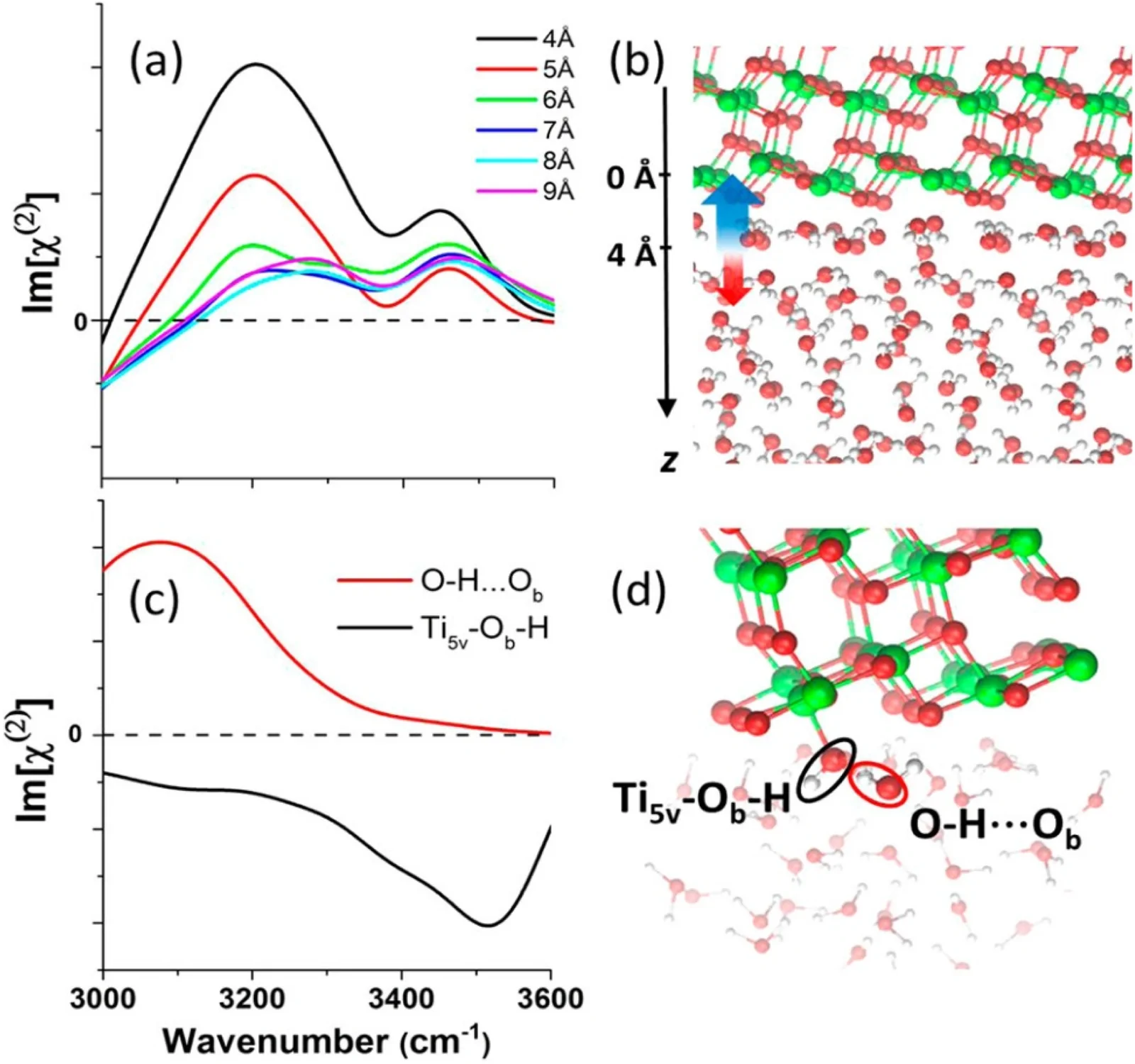
TiO_2_ surface interfacial water structure. (a) Depth-dependent Im[*χ*^(2)^] spectra of AIMD simulations. (b) View of the TiO_2_ and water interface shows how water orients. (c) SFG response of contributions of chemisorbed hydroxyl groups and H-bonded O–H groups to the hydroxylated surface. (d) Potential picture of the hydroxylated TiO_2_surface with water molecules. Reprinted with open access permission from American Chemical Society.^56^

The MD simulations predict that the binding energy of the first hydration layer is between 0.75 and 1.1 eV, with simulation times up to 10 ns, which is significantly longer than the picosecond timescales of bulk water.^100–102^ The interfacial viscosity becomes substantially larger than the bulk fluid viscosity, creating a viscous drag force that hinders ion and signalling protein transport to the implant surface.^103,104^ One discrepancy observed in experimental characterization is that zeta-potential values predicted by Poisson–Boltzmann are typically a factor of 30% to 50% lower than experimental models of smooth nanomechanical surfaces.^105^ This inconsistency cannot be explained by measurement error alone, but may be explained by a fundamental physical process that is associated with the shear plane of the electric double layer (EDL). The fluid flow is approximately zero at the solid surface according to the Poisson–Boltzmann theory. However, the hydration shield, a complex of chemically bound and physically trapped water, moves along with the particle. This shear plane movement causes a drop in the immobilized liquid layer, which is not described by the standard models.^106,107^ To initiate osseointegration, bone-repair proteins like fibronectin or BMP-2 must disrupt this quasi-solid layer, displacing the attached water molecules, which occurs through the dehydration penalty. The associated energy cost has been reported to be approximately 0.2–0.5 eV per adsorbed protein molecule.

From a thermodynamic perspective, protein adsorption is only favourable when the entropy gain of bound water molecules released into the bulk fluid offsets the enthalpy cost of breaking the water bonds. As discussed in Section 3.1, the SIE errors in DFT can affect predictions of surface charge density and the hydration layer behavior, which leads to inaccurate modelling of biomineralization kinetics and protein adsorption. Besides, the alignment of the water dipoles in this barrier creates a local electric field that can reach a magnitude of up to 10^7^–10^8^ V m^−1^. This strong field helps screen the outward charge of the implant; cells sense not the metal itself but the organized water shell surrounding it. This means that the effective design of a hydroxyapatite-coated heterojunction will depend strongly on controlling surface defects that disrupt hydration continuity, allowing the mineral ions to penetrate the barrier and start mineralization.^108^

### 5.2. Competitive ion adsorption thermodynamics

The kinetic and thermodynamic competition governing the formation of mineralized HA–TiO_2_ heterojunction is based on the Langmuir–Hinshelwood model, where Ti–OH surface sites are the main reaction sites. Although the physics of band offsets and space-charge layers was addressed in the previous section, these two electronic properties directly modulate responsible for the modulation of the electrostatic attraction between phosphate ions (PO_4_^3−^) and calcium ions (Ca^2+^).^109^ These two electronic characteristics are described by the Grahame equation relating surface charge density to interfacial potential. The DFT-predicted phosphate adsorption energy is −3.2 eV, which is theoretically high enough to remove the water molecules.^110,111^ However, this displacement is physically hindered by the hydration barrier as well as the steric barrier of the large phosphate tetrahedron approaching the structured water layer. Interfacial strain energy arises between the hexagonal HA lattice and the tetragonal TiO_2_ substrate, competing with the chemical bonding energy.^112,113^ Mathematically, net adsorption energy may be defined by eqn (1):

1 Δ*G* = Δ*G*_adsorption_ + Δ*G*_strain_

where Δ*G* is the net adsorption energy, Δ*G*_adsorption_ is the chemical adsorption energy, and Δ*G*_strain_ is the interfacial strain energy. When the strain energy is larger than the chemical bond energy, the system prefers to grow into an island form (Volmer–Weber mode) instead of a continuous coating, which is the cause of the delamination risk.^114^ This is addressed by deprotonating surface adsorbates on HA, *e.g.* H^+^ and OH^−^, by a reaction controlled by the multisite complexation model. This facilitates later adsorption on TiO_2_ oxygen vacancies, forming an active chemical pathway, by means of proton hopping (Grotthuss mechanism).^115,116^ Doping with strontium (Sr^2+^) also modifies the surface physics by changing the density of states at the Fermi level. The impact of Sr^2+^ doping is that the electronegativity gradient at the interface is increased in a favourable direction, altering the competition for mineralization. This change decreases the desolvation energy needed to remove the primary hydration shell (first 6–8 water molecules that enclose the ion) of Ca^2+^. Sr^+^ doping reduces this energy barrier to below the normal +20 kcal mol^−1^ range, and thus ions enter the first hydration shell of the TiO_2_ implant with higher frequency.^117,118^

### 5.3. Nanoscale confinement effect on TiO_2_ nanotubes

Nanostructuring through the fabrication of TiO_2_ nanotubes (TNTs) is an effective physical approach to mitigate lattice-mismatch and strain-related limitations.^97,119^ TNTs may disrupt the formation of a continuous, “locked” hydration layer by creating non-uniform electric fields at the nanotube opening, introducing high-curvature nanoscale structures.^120,121^ This geometric perturbation prevents the creation of an ice-like lattice by causing an imbalance in the hydrogen bond angle due to curvature. The surface free energy, which has been reported to increase by 45 mJ m^−2^ on flat titanium, is more than 75 mJ m^−2^ on nanotube walls that are the high-affinity sites for ion adsorption. In the dimensions of the nanotube (15–100 nm), the Gibbs–Thomson effect guarantees that the supersaturation of ions may locally reach up to ∼300% relative to that of the bulk fluid.^122^ This is because the chemical potential of the fluid contained within the confined nano-channel is greater than that of the bulk reservoir, which essentially pushes ions contained in the liquid phase to the solid HA phase.^123–125^ In addition, the Laplace pressure (Δ*P* = 2*γ*/*r*) present within these nanotubes produces a local pressure gradient that promotes the capillary rise of physiological fluids. This force helps in the rapid transport of Ca^2+^ and PO_4_^3−^ ions into the depth of the nanotubes, where they are less exposed to the shear forces of blood flow. The geometric catalysis of this nanoscale confinement helps in decreasing the radius of a critical nucleus (*r*).^125,126^ TNTs reduce the energy barrier needed during nucleation, and the induction time of hydroxyapatite can be made several days shorter than in the absence of the nanotube, resulting in the paradigm of biological fixation being achieved within the critical clinical window of early implant stability.^127^

## 6. Dynamic interface degradation and ion transport

The introduction of the HA–TiO_2_ heterojunction into the physiological environment is not limited to static structural support but constitutes a dynamic bio-physical interface of energy transduction and mass flow that determines the long-term viability of the implant. Whereas the above section was concerned with the issue of aqueous barriers and competitive adsorption on the nanoscopic scale, this section focuses on the functionality of the material under physiological load and its time-controlled degradation.^11,116^ To assess the switching between passive and active chemical binding, this section is based on the piezoelectricity of the hydroxyapatite phase and the complex kinetics of osteogenic ion migration. The main assumption behind this section is that a smart biomaterial should be an electromechanical transducer. These phenomena may be modelled by the Nernst–Planck framework and dipole dynamics, which allow quantification of the ability of the material to transform mechanical energy, provided by patient movement, into cellular instructions. Such bio-physical dialogue may influence fibrous-tissue formation and contribute to the implant becoming part of the living skeletal system.^129,130^

### 6.1. Piezoelectric response and surface potential modelling

Experimental measurements on bulk hydroxyapatite crystals have established that the OH^−^ dipole flip in the hydroxyapatite (HA) lattice is the basis of bio-physical signalling between the implant and the bone tissue. The mechanism is still a foundational pillar related to the electro-active behavior of the material, and the implant can thus act as a bridge between the prosthesis and the living tissue. The addition of TiO_2_ into the composite matrix is a deliberate design strategy to maintain the dielectric characteristics of HA. This is necessary to ensure a piezoelectric coefficient (*d*_33_) that is consistent with natural cortical bone, which has been experimentally measured as a coefficient of about 0.7 pC N^−1^.^11,131^ It is well established that the orientation of OH^−^ dipoles along the *c*-axis forms a non-centrosymmetric structure that is highly sensitive to external pressure in crystalline HA.^130^ The mechanical stress (*σ*) acting on the prosthetic interface when a patient is subjected to load-bearing activities is predicted by computational models to be transformed into a localized potential (*φ*) through the piezoelectric effect. This relationship is described by the following governing eqn (2):2
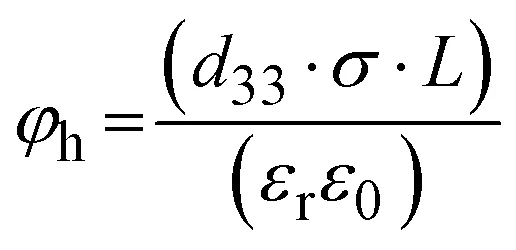
where *φ*_h_ is the piezoelectric potential generated at the interface, *d*_33_ is the piezoelectric coefficient, and *σ* is the mechanical stress applied. *L* is the thickness of the coating, *ε*_r_ is the relative permittivity, and *ε*_0_ is the permittivity of vacuum. Eqn (2) describes the conversion of mechanical stress into localized electrical potential through the piezoelectric effect. Fig. 7 shows a hierarchical bone structure with a focus on the main sources of piezoelectricity. Although the piezoelectric potential and the stiffness of the mineral are shown by the reorientation of OH dipoles in the HA, the aligned collagen fibrils act as the principal biological source of piezoelectricity.^132^ This hierarchy is capable of providing bioelectric sensing of bone remodelling and repair. DFT and continuum models predict that the coating produces potentials on the order of millivolts (mV) under normal physiological loads of 2–20 MPa.^78,133^ These transitory electrical currents are important as they exceed the threshold necessary to alter the zeta potential of the surrounding fluid, and as such, affect the movement of the charged signalling proteins. The proposed mechanism suggests that these millivolt-scale potentials, in physical terms, trigger the opening of the voltage-gated calcium channels (VGCCs) in the adjacent osteocytes and osteoblasts. This promotes rapid influx of Ca^2+^ ions into the cell, which then upregulates the expression of osteogenic genes.^14,134^ The conceptual model proposes that the interface can be regarded as an electro-mechanical system that can simulate the natural Wolff's law of bone remodelling. In this way, the biological fixation paradigm is achieved by active signalling rather than by physical presence alone.^124,131^*In vivo* validation of this procedure remains an active area of research and clinical investigation.

**Fig. 7:**
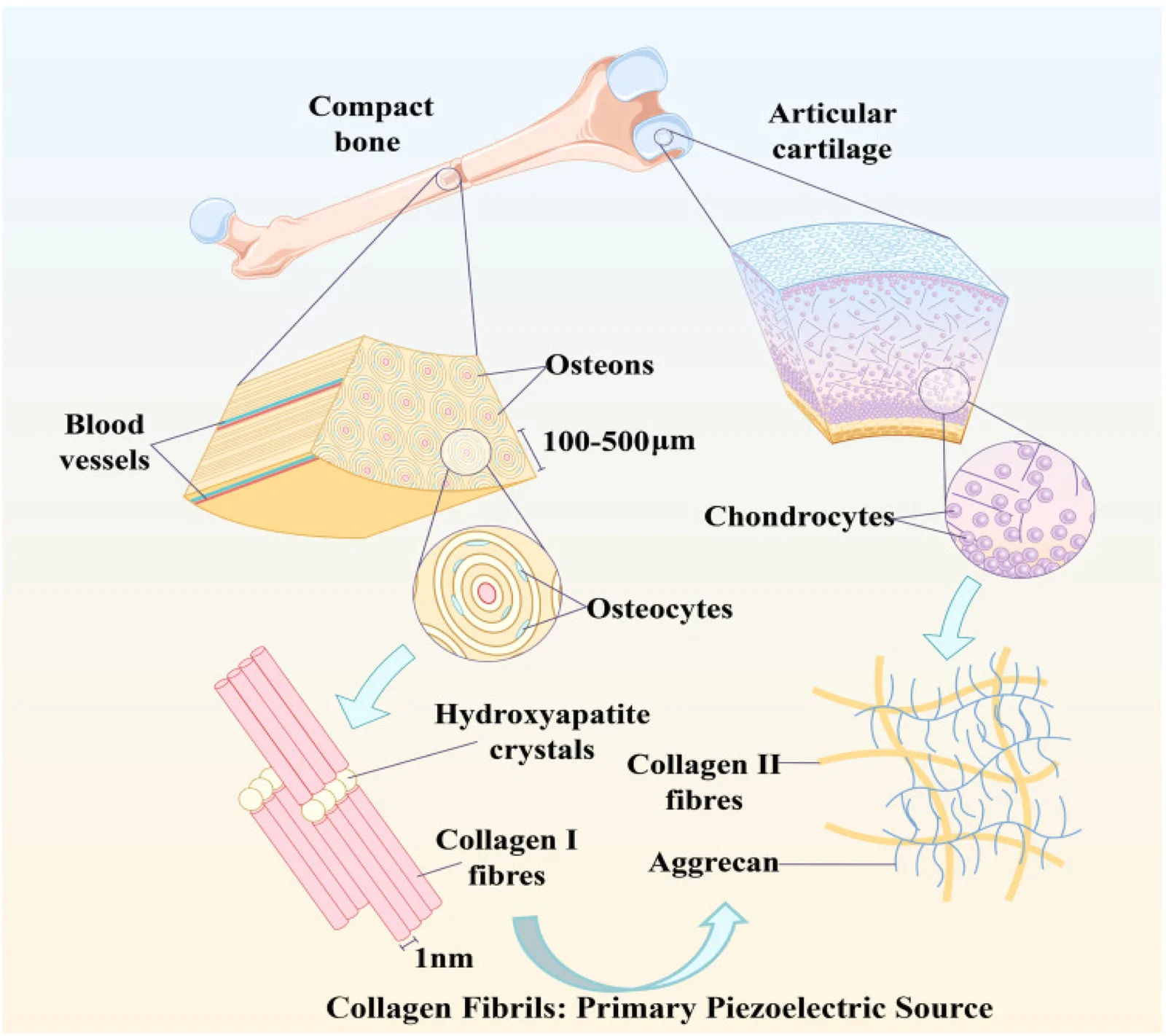
Bone structure hierarchy in which collagen fibrils produce piezoelectricity and with hydroxyapatite to give mechanical rigidity. Reprinted with open access permission from Dove Medical Press.^132^

### 6.2. The Nernst–Planck framework and selective ion migration kinetics

The migration of ions and controlled degradation determine the long-term therapeutic and structural stability of the HA–TiO_2_ heterojunction.^135^ This is modelled by the Nernst–Planck eqn (3), which describes the flux (*J*) of ions subject to a concentration gradient (diffusion) and electroactive surface potentials (drift):3
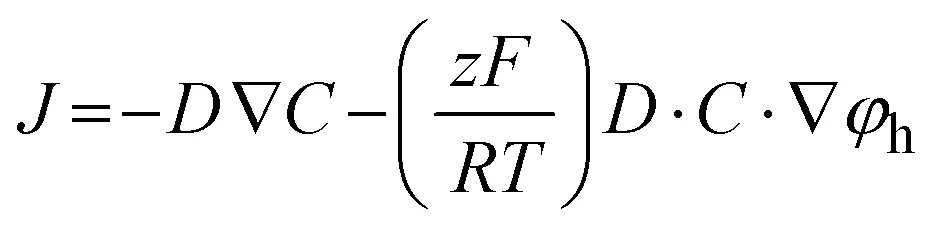
In this case, *D* is the diffusion coefficient, *z* is the ion valence, and *F* is the Faraday constant. Whereas, *R* is the gas constant, *T* the absolute temperature (310 K at physiological conditions), *C* the ion concentration and *φ* the electric potential.^37^ In this instance, the piezoelectric potential gradient is ∇*φ*_h_. The compiled data presented in Table 4 define the overall kinetic pathway for converting a hydrated TiO_2_ surface into a mineralized hydroxyapatite (HA) coating. The tabulated diffusion coefficients correspond to free-solution values at 25 °C;^136^ interfacial transport through the confined hydration layer at physiological temperature (37 °C) is expected to be slower, and the Table 4 values are used here only as an order-of-magnitude reference. The migration of the osteogenic ions is not uniform but a highly selective movement based on the fact that selective movement is determined by the different diffusion coefficient and activation energy barrier (*E*_a_) of each specie. It is found that the Ca^2+^ ions have a respectable mobility of 0.79 × 10^−9^ m^2^ s^−1^. This is mainly because interfacial hopping enables small divalent cations to traverse the interfacial hydration layers at a reduced energy penalty of ∼19 kJ mol^−1^.^136^ Rapid accumulation of Ca^2+^ forms a positively charged precursor layer, which is vital for subsequent electrostatic binding of phosphate groups. Conversely, the rate-limiting step in the biomineralization process is the migration of phosphate. Phosphate ions migrate much more slowly than calcium ions, as their diffusion coefficient is lower and their activation barrier is higher.^48^ This difference can be explained by the fact that they possess bulky tetrahedral geometry and dependence on ligand exchange with surface-bound water molecules.

**Table 4:** Ion Diffusion rates and their biological effects

Ionic species	Diffusion coefficient m^2^ s^−1^ (at 25 °C)	Activation energy	Migration pathways	Biological impacts
Calcium (Ca^2+^)	0.79 × 10^−9^	∼19 kJ mol^−1^	Vacancy-mediated jumping	Stimulates osteoblast adhesion and fixes the primitive mineral framework^136,145^
Strontium (Sr^2+^)	0.79 × 10^−9^	20.4 kJ mol^−1^	Isomorphous substitution	Stimulates bone growth and suppresses bone renewal (osteoclasts)^140^
Phosphate (PO_4_^3−^)	0.55 × 10^−9^	22.1 kJ mol^−1^	Ligand displacement	Stoichiometric HA crystallization rate-limiting substrate^137^
Hydroxyl (OH^−^)	2.10 × 10^−9^	12.8 kJ mol^−1^	Cooperative proton hopping	Maximizes the interfacial acidity to fasten apatite precipitation^37^

The kinetic variables in Table 4 indicate that this is a fundamental control of the stoichiometry of the growing layer of HA (ratio of 1.67) by the diffusion lag of Phosphate.^6,138^ Moreover, it is further augmented by the addition of strontium (Sr^2+^), which leads to therapeutic kinetic modulation. A design strength is the kinetic profile of strontium, whose diffusion coefficient (∼0.79 × 10^−9^ m^2^ s^−1^) is closely comparable to that of calcium.^136^ The burst release effect of unstable interfaces is inhibited by an increased ionic radius of Sr^2+^. Rather, it promotes a regulated-release process that provides sustained biochemical stimulation of osteoblasts, thus enhancing long-term structural integrity of the bone-implant interface. Perhaps the most notable point in Table 4 is that hydroxyl ions (OH^−^) have exceptionally high mobility. In contrast to the diffusion of an equivalent mass-transport-limited diffusion, limited by the diffusivity of the medium, the ions apply the Grotthuss Mechanism (cooperative proton hopping), as reported.^37,139^ This extremely high operative speed is an essential part of physiologic control, nearly instantaneous neutralization of acidic excretions at the tissue–implant interface. The high rate of ionic exchange by providing local pH homeostasis also ensures that the thermodynamic window allowing HA precipitation is open, which is in effect a kinetic catalyst to the whole process of osseointegration.^37,140^

Diffusion coefficients of Ca^2+^ and PO_4_^3−^ ions moving out of the coating into the surrounding periprosthetic fluid are of a similar order of magnitude, ∼10^−9^ m^2^ s^−1^ (Table 4). The very low activation enthalpy of TiO_2_ at low temperatures below 100 °C allows the material to be released during the first crucial stage of the bone consolidation. Nevertheless, to avoid grain sublimation or mechanical breakdown, the rate of dissolution should be carefully adjusted to about 1 µm day^−1^. One of the major physical factors involved in this migration process is the tortuosity (*τ*) introduced by nano-structuring.^141^ The tortuosity of TiO_2_ nanotube (TNT) architectures is usually in the range of 1.2 to 1.5, which directly limits the free diffusion of ions and in effect, prolongs the life of the mineral reservoir. This slow release ensures that the local calcium concentration does not exceed the optimal osteogenic concentration (2.5 to 5.0 mM), over a period of more than six months.^139,142,143^ Moreover, the chemical glue effect of dopants like strontium also has a dual purpose as it not only stabilizes the HA lattice against early dissolution but is also a catalytic agent, which enhances the local supersaturation of phosphate ions at the nanotube interface.^137,140^ This ensures a continuous biomineralization process that meets the macro-scale requirements of mechanical resilience and clinical success.^144^

## 7. Multiscale modelling roadmap for bio-ceramic interfaces

Preventing fibrous encapsulation and maintaining implant stability remain major long-term challenges for biomaterials engineering. The solutions to these challenges are a systematic transition from laboratory-based empirical observations to multiscale physics-based predictive solutions. A proposed modelling framework is described that couples the atomic-level quantum mechanical description of the material to the macroscopic description of clinical behavior, offering a computational path between the different temporal and spatial scales of the biomaterial sciences. Several individual links have been demonstrated for TiO_2_ or HA separately, shown in Table 5, but a validated end-to-end HA–TiO_2_ model has not yet been reported. The framework is therefore a research roadmap. The design of HA–TiO_2_ heterojunctions can be changed to be a predictive engineering science instead of a trial and error process by using basic physics in an integrated platform.^146^ Several gaps must be bridged to link clinical outcomes to computational physics. Section 8 benchmarks the relevant methods in detail. In addition, mean-field Poisson–Boltzmann treatments of zeta potentials usually do not consider the molecular solvent effects; however, explicit-solvent simulations or neural-network MD can be employed to address this issue, and the free energy of the interfaces can be used as a test for the validity of the treatment. Lastly, the lattice mismatch strain at the HA–TiO_2_ interface may contribute to coating delamination, and it is necessary to have multiscale finite element and phase-field modelling of this strain and its adhesion properties, which can be validated by empirical measurements of the interfacial shear and adhesion strength.^147,148^ The mechanical lifetime and degradation mechanisms of the HA–TiO_2_ heterojunction could in future, be anticipated using this multiscale roadmap, which explains the connection between quantum-scale phenomena and clinical performance, as summarized in Fig. 8.

**Table 5:** Proposed multiscale parameter-transfer links for HA–TiO_2_ interface modelling, identifying the parameter transferred, receiving model, available validation, and whether each coupling is demonstrated, partly established, prospective, or a future objective

Link	Parameter → receiving model	Available validation	Status	Reference
DFT/HSE06 → MLIP	Energies and forces → neural-network potential	DNN potentials for water on rutile, O-vacancy diffusion in rutile, OH reorientation in HA	Demonstrated for TiO_2_ or HA separately, not for HA–TiO_2_	149
MLIP-MD → mesoscale	Free energies, diffusivities → phase-field/kMC	None found for HA–TiO_2_	Prospective	38
DFT adhesion energy → continuum	*W*_ad_ → interfacial fracture/FEM	Framework exists; no HA–TiO_2_ comparison with shear/scratch tests	Partly established	60
Table 4 *D*, *E*_a_ → Nernst–Planck	*D*, *E*_a_ → ion–release profile	Not validated against ICP-MS dissolution data	Prospective	136
*d*_33_ + stress → potential → cell response	Eqn (2) → mechanotransduction	Sec. 6.1 already says *in vivo* validation is open	Prospective	78
All scales → osseointegration/longevity	—	No validated model	Future objective	—

**Fig. 8:**
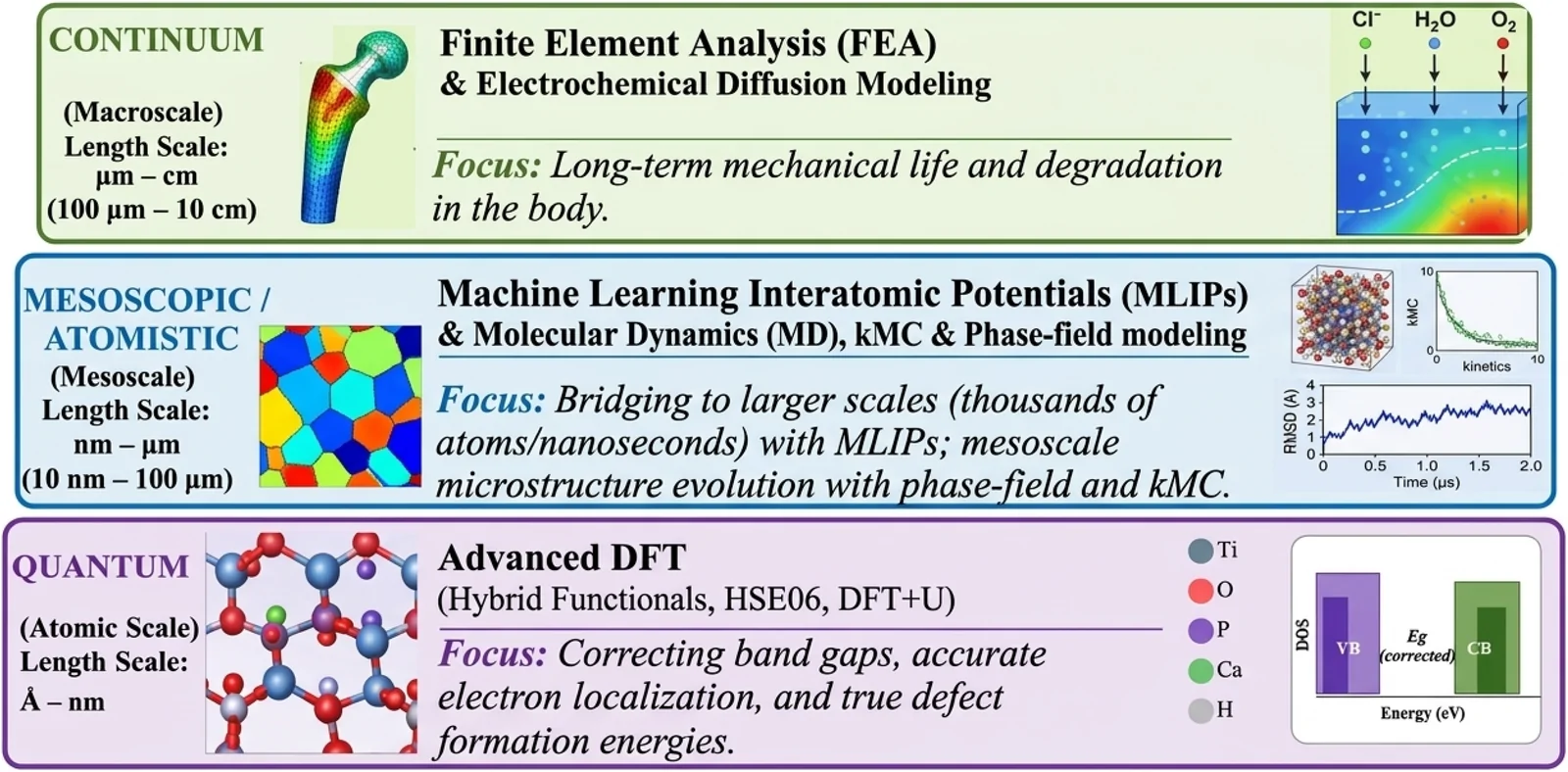
Proposed hierarchical computational roadmap linking quantum accuracy to clinical performance for HA–TiO_2_ interfaces.

The multiscale framework presented here provides a self-consistent approach for connecting electronic structure calculations to clinical outcomes. On the quantum scale, DFT, HSE06, and GW calculations provide fundamental parameters such as the band gap, defect formation energy, mechanical character, and charge transfer properties.^25,30^ These parameters are utilized in atomistic simulations, such as MD and Machine Learning Interatomic Potentials (MLIPs), which provide insights into hydration dynamics, ion transport, and nucleation kinetics.^37,38,149^ Mesoscale models (phase-field and kinetic Monte Carlo) use these inputs to simulate interface evolution and corrosion kinetics.^150^ Further, continuum models convert these predictions to mechanical stability and implant performance metrics.^147,148^ This hierarchical workflow is projected as a roadmap for future quantitative forecasts. Now, only the quantum-to-atomistic parameter transfers (*e.g.*, DFT/HSE06 band gaps and defect energies to MD/MLIP) have been established and benchmarked. Clinical outcomes such as implant longevity require an effectively validated coupled HA–TiO_2_ model and are not yet quantitatively predicted by this framework. This integrated approach enables understanding of how quantum-level defects in HA–TiO_2_ interfaces influences mesoscale ion transport and consequently on clinical performance, as illustrated in Fig. 9.

**Fig. 9:**
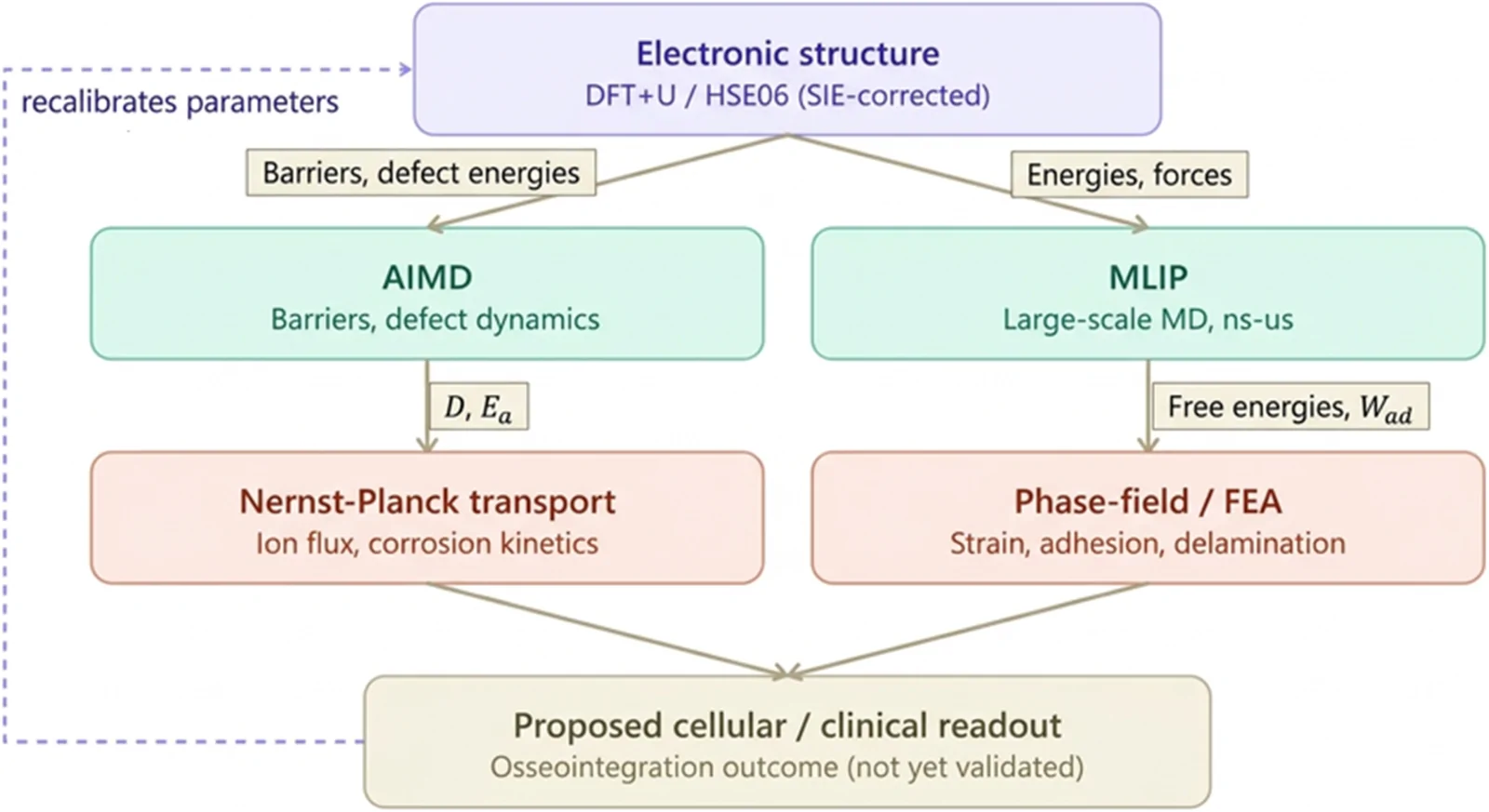
Proposed self-consistent multiscale framework for HA–TiO_2_ interface modelling, showing parameter transfer from quantum to clinical scale.

### 7.1. Development towards machine-learning interatomic potentials

The application of MLIPs in the simulation of the HA–TiO_2_ heterojunction is a major advance in overcoming the long-standing scale gap between quantum-accurate simulation and biointerface activity of interest.^151,152^ Although valuable to the study of interfacial energetics, such as the energy of V_o_ formation to surface termination, oxygen hydroxyl group energetics and barrier to proton transfer (*e.g.*, that between water and oxygen) to < 0.5 eV and timescales on the order of 10–100 ps.^153–155^ DFT has had comparatively poor practical utility because its system sizes are limited to ∼10^3^ atoms and of its picosecond timescale cannot provide sufficient statistical sampling of the pathway for nucleation, defect migration, and strain relaxation that control the development of hydroxyapatite (HA) on TiO_2_ surfaces.^156,157^ The weakness of these methods is solved in MLIPs that create DFT-level potential energy surfaces at a fraction of the cost.^158,159^ This, in practice, permits molecular-dynamics simulations of atoms and timescales of microseconds, or many more orders of magnitude of phase space than the accessible phase space of AIMD. This is transformative in the case of the HA–TiO_2_ interface.^160^ MLIP models can be used to perform large-scale simulations with varying lattice mismatches (typically 2–8%, depending on the HA model and TiO_2_ polymorph), elastic relaxation length and heterogeneous surface terminations (*e.g.*, hydroxylated, reduced, or defect-rich TiO_2_). These factors are known to determine HA orientation, crystallinity, and adhesion strength, yet they are largely accessible only through direct atomistic modelling. Most importantly, it is possible to study the mechanisms of non-classical nucleation directly using MLIPs.^44,95^ Emerging experimental evidence indicates that the HA formation can be proceed *via* either the amorphous calcium phosphate precursors or the prenucleation clusters assisted by the presence of surface hydroxyl groups. The latter is required in the scale of the system and the time span of the simulation to observe such stochastic, metastable intermediates, which are very rare and very sensitive to local chemical fluctuations.^157,161^

Although the transformative potential is great, several important limitations of MLIPs must be considered. First, MLIPs are data-driven models with data interpolation properties, and the diversity of training sets strongly influences model performance. The resulting potential can give inaccurate predictions for untested conditions if the training data are not sufficiently representative enough of the configurational space relevant to the prediction, such as different surface terminations, defect configurations, water molecules presence and distribution, and ion distribution.^162^ This is critically challenging for HA–TiO_2_ interfaces, where the chemical environment is complex and highly variable. Second, the chemical diversity of the interfaces to which the results will be transferable is still hard to accomplish. Generalization of the trained compositions or phases to other environments (*e.g.* different HA stoichiometries, doping concentrations and mixed oxide interfaces, are considered prerequisites for MLIPs related to other HA materials. The PES of a chemically complex interfaces) is high-dimensional due to which the systematic extrapolation errors take place when atomic configurations are encountered outside of the range of configurations learned by the model. Last, the electronic structure phenomena related to charge transfer, polarization, dynamic change in charge distribution and electronic excitations cannot be considered a part of the description of MLIPs.^163^ The electronic effects play key role in the understanding of the redox chemistry, the band alignment, the formation of the polaron and photocatalytic processes at the HA–TiO_2_ interface. Therefore, MLIPs suffer challenges to predict properties needing a quantum mechanical description, such as electronic structure methods.^164^

MLIP-based MD can statistically determine nucleation rates, sizes of critical nucleus sizes, and the selective orientations of numerous structures under various pH, ionic strength and temperature conditions. Despite these advantages, there are important methodological challenges as far as the use of the MLIPs at bio-ceramic interfaces is concerned.^149^ MLIPs have an exclusive strategic position from a multiscale modelling perspective. They allow the extraction of mesoscale descriptors, interfacial free energies, growth anisotropy coefficients, a diffusion constant of defects, and strain-dependent adhesion energies, which can all be generalized into phase-field and continuum models of coating formation and bio-integration space systematically. This paradigm of hierarchical workflow in quantum chemistry could provide a route in a quantitative route to clinically significant surface workflow measures.^165^

Collectively, the paradigmatic change of the computational model of HA–TiO_2_ heterojunction to MLIP is not only an improvement in computational models but also a shift in the theoretical analysis of HA–TiO_2_ heterojunctions. Not only can MLIPs statistically and quantum-mechanically simulate experimentally relevant length and time scales, but they are also required for predictive design of biomimetic implant surfaces and rational optimization of the osseointegration behavior.^79,147^ However, the successful application of MLIPs to HA–TiO_2_ interfaces will depend on addressing their important drawbacks through cautious training data selection, systematic validation against reliable electronic-structure calculations, and integration with balancing quantum-mechanical methods for properties beyond the scope of classical force fields.^162^

## 8. Benchmarking electronic structure and multiscale methods

There are several approaches used to accurately model the HA–TiO_2_ interface, which have direct implications for the properties mentioned in the preceding sections, such as band gaps, V_o_ formation energies, and redox behavior; these must be balanced against computational cost. The most affordable approach is the standard gradient approximation (GGA) functional, for example, PBE, which significantly underestimates the band gaps of TiO_2_ polymorphs (anatase has 3.2 eV and rutile has 3.0 eV), and thus shifts the V_o_ formation energies to unrealistically low values (2.0–2.5 eV). Delocalization errors are corrected far better by hybrid functionals such as HSE06, albeit at substantially higher computational costs, bringing the V_o_ formation energies closer to the measured values by electron paramagnetic resonance (EPR). The on-site Coulomb-correction method DFT+U is a cheaper alternative to hybrid functionals for localized 3d/defect states, but its accuracy is U-dependent. So, it is not universally equivalent to HSE06. Band gaps, redox potentials, and adsorption energies should be benchmarked against HSE06 or experiment for each system. Many-body perturbation theory (GW) and machine-learning-corrected DFT can further minimize SIE-related error for certain applications, such as determining the positions of quasiparticles in band offsets, but the present costs hinder their practical use in large-scale biomaterial simulations at this time.^166^

The methodological criteria should include accuracy, computational cost, accessible system sizes, timescales (scalability), and appropriate level of theory for making accurate predictions related to HA–TiO_2_ interface problems. Table 6 summarizes these criteria, outlining the methodology, computational cost, practical scales, and limitations for modelling of HA–TiO_2_ interfaces.^167^ The accuracy in reproduction of the experimental band gaps, V_o_ formation energies, and adsorption energies by different levels of theory is also elaborated. The scalable predictions on heterostructures is important, as it indicates the modelling of large system size at large simulation time scale *via* using the specific method. These criteria provide guidelines for selecting a robust framework that helps identify the appropriate method for meeting planned research objectives. Thus, considering the expensive calculates related to the large interface configurations of the heterostructure, despite its underestimating energetic character, we propose GGA/PBE is suitable for initial screening. Subsequently, accurate determination of band gaps, V_o_ formation energies, defect states, and interfacial charge transfer character should be performed at the HSE06 or DFT+U level of theory.^30^ HSE06 yields higher accuracy, but at a higher computational cost, while DFT+U is a more economical approach despite critical decisions in setting Hubbard corrections. The benchmarking and spectroscopic validation of the band offsets are recommended to be obtained by GW or ML-corrected DFT, although these are costly and usually feasible only for small systems. For long-timescale simulations of nucleation, defect migration, and strain relaxation, MLIPs are the particularly suitable approach, providing accuracy relative to reference DFT data, and microsecond time scales to calculate systems of about 10^3^–10^6^ number of atoms.^160^ It is, however, important to note that MLIPs are unable to describe charge transfer, polarization, or electronic excitations, and should be used in conjunction with any explicit electronic-structure methods when any of these properties are taken into account. This decision-making framework offers a step-by-step guideline for researchers to choose computational methods for modelling HA–TiO_2_ interfaces, depending on their research interest and computational power.

**Table 6:** Comparison of electronic-structure and multiscale modelling methods for HA–TiO_2_ interfaces

Method	Accuracy criterion	Relative cost	Practical scale	Best use at HA–TiO_2_ interface	Main limitation	Ref.
PBE/GGA	Good structural/energetic trends; systematically underestimates many semiconductor band gaps	Low	Large relative to hybrid methods; short AIMD	Initial interface screening, structure relaxation, adsorption trends	Self-interaction/band-gap and defect-state errors	25
DFT+U	Improves localized Ti 3d/defect description; U-dependent	Low–moderate	Moderate	TiO_2_ defects, localized states, redox energetics	Results depend on U; not universally equivalent to HSE	30
HSE06	Generally improved band gaps/defect electronic structure	High	Small-moderate	Benchmark electronic structure, selected defect/interface models	High computational cost	35
GW	Quasiparticle-level electronic structure	Very high	Small	Quasiparticle energies and high-level band alignment	Very expensive; impractical for full biomaterial models	25
MLIP	Accuracy determined by energy/force errors on representative validation set	Low inference cost after training	Large → very large; timescale strongly hardware/model dependent	Long MD, nucleation, defect migration, strain relaxation	No explicit electronic structure; extrapolation risk	160

All of these electronic-structure methods cannot achieve the length and time scales needed to study nucleation processes, defect migration, and strain relaxation processes at the HA–TiO_2_ interface, as conventional DFT is limited to ∼10^3^ atoms and the picosecond time scale. MLIPs overcome this limitation by maintaining the quality of the potential energy surface achievable by DFT, but at a much lower cost, making them accessible for simulation times of up to microseconds, which is several orders of magnitude more than what can be achieved when using AIMD. This renders MLIPs particularly suitable for statistically sampling nucleation kinetics, defect migration, and strain relaxation pathways. The major drawbacks of these models are that they are necessarily data-driven and interpolative; the degree of accuracy is limited by the diversity of the database, and they only describe total energies and forces; therefore, they cannot explicitly describe any of the processes mentioned above, such as charge transfer, polarization, or electronic excitation. Therefore, MLIPs should be treated as a complementary tool, rather than a substitute for explicit electronic-structure methods; for properties such as redox chemistry, band alignment, or photo-activation processes, DFT/DFT+U methods are still required. In Table 6, this accuracy-cost landscape is summarized precisely for multiple methods being used.

## 9. Critical assessment and physics-based benchmarking

Translation of the computational models from academic research to the clinical practice requires rigorous evaluation of modelling reliability and cross-institutional reproducibility.^148,168^ A hierarchal parametrization is used to integrate the electronic structure calculations with clinically relevant outcomes. The fundamental materials properties such as the band gap, defect formation energies and charge transfer properties obtained from DFT and HSE06 calculations are routinely utilized to describe the electrochemical stability of the implant surface at the quantum level. The band gaps of TiO_2_ polymorphs obtained at PBE and HSE06 levels of theory set the electronic structure landscape to describe the charge transfer and surface reactivity at the implant interface.^25^ The formation energy of oxygen vacancies, calculated by hybrid functional, predicts the tendency of defect formation to establish the subsequent production of reactive oxygen species (ROS).^30^ Properties directly related to corrosion resistance include oxidative stress and inflammation at the tissue–implant interface which are linked to vacancy concentrations.^63^ Calculations of adhesion energy using DFT and DFT-D3 methods control coating stability and delamination resistance under physiological loading conditions. The van der Waals interactions play a major role in the adhesion energy at the a-HA/a-TiO_2_ interface.^12,60^ MD and MLIP simulations provide hydration-dynamics insight,^149^ which, combined with the ion-transport data compiled in Table 4, shed light on biomineralization dynamics and Ca^2+^/PO_4_^3−^ ion exchange at the implant–bone interface. These mesoscale phenomena are ultimately lead to osseointegration outcomes and implant longevity. The multiscale framework offers a proposed roadmap towards quantitative predictions of clinically relevant performance metrics such as corrosion rates, coating adhesion strength, and bone integration potential, which could, in principle, be obtained from first-principles calculations once the roadmap is fully validated—potentially offering a computational route to rational implant design. Table 7 summarizes the main computational bottlenecks and proposed solutions.

**Table 7:** Computational bottlenecks and proposed solutions

Models	Technical limitations	Proposed solution	Key validation metric	Ref.
Electronic self-interaction	The error of conventional DFT in self-interaction leads to redox potentials and band gaps being inaccurate	Hybrid XC functional (HSE06, B3LYP) or DFT+U corrections	Adsorption energies, density of states and defect creation energies	169 and 170
Interfacial electrostatics (zeta potential accuracy)	Discrete molecular solvent effects are ignored by the mean field Poisson–Boltzmann (PB)	Neural network MD (NNMD) with explicit solvation	Distributions of ion adsorption and interfacial free energy profiles	149 and 171
Temporal scale limitations	Cannot predict accurately the real-time nucleation (millisecond) and MD is restricted to picosecond	Machine learning interatomic potentials (MLIP)	To obtain the larger picture namely, nucleation statistics, phase-transformation pathways and long-time kinetics, other approaches are heavily needed	172
Lattice mismatch strain	Lattice mismatch generates spatially dependent stresses, which are difficult to measure at one scale	Phase-field informed coupling and multiscale FEM to capture adhesion phenomena	Shear strength and interfacial adhesion energy	148 and 168

The 30% to 50% difference in measured zeta potentials between the fitted Poisson–Boltzmann models and the measured zeta potentials may indicate an underlying deficiency in the knowledge of the immobilized liquid layer at the solid–liquid interface. To resolve this issue, standardized physics-based benchmarks of TiO_2_ V_o_ concentrations and HA adhesion energies are proposed. To establish clinical validity of the predictive models, predictions should be statistically correlated with the standardised experimental measures, including nano-scratch testing, X-ray photoelectron spectroscopy (XPS), and adhesion force measurements. Further, to enable the predicted characteristics of the materials, including corrosion resistance and interfacial bonding strength, to be consistent and reliable across various research groups.^169,170^ Finally, the development of these standards may help realise the longer-term vision of a personalized HA–TiO_2_ heterojunction, adjusted to the bone density of a particular patient and his/her physiological load, could potentially be determined and confirmed even prior to clinical implementation once adequately validated.^147^

## 10. Summary

The use of multiscale modelling to enable efficient predictions for Hydroxyapatite-TiO_2_ bio-interface coating design before clinical orthopedic implantation is thoroughly described. This review highlights key approaches such as DFT+U, hybrid functionals (HSE06), and MLIPs, which address self-interaction problems. The issues related to the electronic structure calculations, which hinder their capability for predicting the surface reactivity for TiO_2_, formation energies of oxygen vacancies, and early-stage corrosion behavior of TiO_2_, are elaborated in detail. DFT+U and hybrid functional corrections yield values closer to experimental measurements for both TiO_2_ polymorphs bandgaps and vacancy formation energies; but the values are slightly higher than those of the experimental measurements. The corrections are required to bridge the computational to experimental gaps, as it directly impact the prediction of oxidative stress, electron transport, and electrochemical stability, which in turn impact implant longevity. The presence of a structured hydration layer at the TiO_2_ surface is a key physical barrier controlling bioactivity at the interface. The binding energy of water molecules is in the range of 0.75–1.1 eV, the structured hydration layer is like ice, and it is a good kinetic barrier for protein adsorption and ion transfer. The rate-limiting step in the biomineralisation involves the migration of PO_4_^3−^ ions (*D* = 0.55 × 10^−9^ m^2^ s^−1^, *E*_a_ = 22.1 kJ mol^−1^), as indicated by the competitive thermodynamics of their migration. The interfacial chemical glue effect of strontium doping reduces formation energy barriers by 12–25%, and enhances adhesion energy, which supports controlled ion release and long-term osteogenic stimulation. The spatial and temporal scale separation needed for aiming a machine-learning interatomic potential at bridging relevant length and time scales related to physiological processes ranges from 10^3^ to 10^5^–10^6^ atoms. However, these MLIPs are high data-consumption interpolative models, which cannot be augmented by various training data sets, and do not explicitly include the effect of charge transfer, polarization, and electronic excitations. Several knowledge gaps are identified which prevent the efficient utilization of this model in the field of implant design predicted values. Experimental validation should involve quantitative correlation between computed V_o_ formation energies and electrochemical impedance spectroscopy and X-ray photoelectron spectroscopy data. Hence, a simulated physiological loading to show adhesion strength, and *in vivo* testing to show long-term performance and osseointegration results is required to make a quantitative correlation with the computed results.

Accurate electronic structure modelling of mesoscale hydration dynamics and ion-transport kinetics, along with machine-learning-assisted simulations, can enable the transition from empirical coating development to the long-awaited predictive biointerface engineering. An important next step to close the gap between quantum-scale and clinical experiments is to undertake systematic experimental validation and benchmarking and cross-validate the computational procedures on the computational side. With the personalized biomimetic design of tailor-made HA–TiO_2_ heterojunction coating for individual patient conditions, the challenges await to be solved and revolutionize load-bearing orthopedic implantology. The proposed multiscale framework has advanced understanding of HA–TiO_2_ interfaces at the quantum level and their relationship to clinically relevant phenomena such as corrosion, coating adhesion, and osseointegration potential. This work sheds light on a roadmap for rational interface engineering and identifies key knowledge gaps, including the need for standardised benchmarks and improved MLIP transferability, guiding future research toward the most impactful directions.

## Conflicts of interest

The authors declare that they have no known competing financial interests or personal relationships that could have appeared to influence the work reported in this paper.

## Data Availability

No primary research results, software or code have been included and no new data were generated or analysed as part of this review.
